# Gender differences and smoking cessation in the Japanese smoking cessation treatment program

**DOI:** 10.18332/tid/128497

**Published:** 2020-11-17

**Authors:** Meng Li, Reiko Okamoto

**Affiliations:** 1 Division of Health Sciences, Graduate School of Medicine, Japan

**Keywords:** gender, smoking cessation treatment, Japan

**Dear Editor,**

We read with particular interest the editorial entitled ‘Using human rights measures to advance tobacco control – Japan and the Committee on the Elimination of Discrimination Against Women’^1^ by Action on Smoking and Health. In Japan, tobacco use has been in a nearly constant decline since 1996 and the decline has been mainly accelerating, whereas the smoking prevalence among adult women has essentially not changed^2^. In addition, previous studies have reported that women are facing a variety of barriers to quitting smoking^3^. Here, we wish to express our concern that women find it more difficult to quit smoking than men in the Japanese smoking cessation treatment (SCT) program.

**Figure 1:**
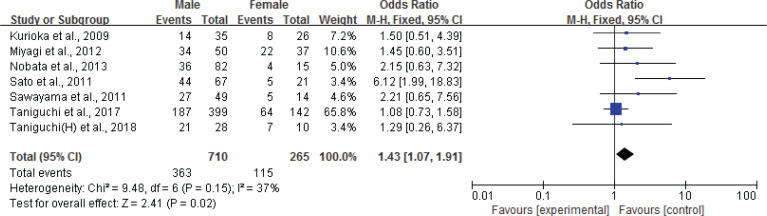
Forest plot of gender differences and smoking cessation at 1 year

To clarify this concern, we conducted a systematic search (Supplementary file) for relevant articles published from 2006 to May 2019, in six electronic databases: Pubmed, CINAHL Plus, Scopus, Web of Science, CiNii Articals, and Ichushi. As a result, 29 articles (n=8199) were included in the analysis. From these studies, we extracted the factors that are associated with smoking cessation, and then conducted a meta-analysis with RevMan 5.3. The analysis (see also Supplementary file) showed that women find it more difficult to quit smoking than men at 12 weeks, with a pooled OR of 1.61 (95% CI: 1.49–1.86, I^2^=26%) among 22 of the studies, which has been reported in ‘Global Tobacco Free Summit TID 15th Annual Conference’^4^. The analysis also showed that women find it more difficult to quit smoking than men at 1 year, with a pooled OR of 1.43 (95% CI: 1.07–1.91, I^2^=37%) among 7 of the studies^5-11^ (Figure 1).

A previous international survey that included the United Kingdom, United States, Canada, and Australia, has reported that the success rate of smoking cessation among women is lower than in men if women do not use any smoking cessation medication, whereas there are no significant differences between women and men if self-selected drugs including nicotine patch and varenicline are provided for use^12^. However, our results showed that women found it more difficult to quit smoking than men, even though smoking cessation drugs including nicotine patch and varenicline were provided for use in the program. This comparison suggests that Japanese women find it more difficult to quit smoking than women in the United Kingdom, United States, Canada, and Australia.

Overall, gender differences and smoking cessation in the program may correspond to discrimination against women in Japan. To eliminate the discrimination, more effective strategies such as providing financial support are needed to help women quit smoking in the future.
