# Supplementary material for: Gender differences and smoking cessation in the Japanese smoking cessation treatment program
Source: Tob Induc Dis. 2020 Nov 17;18:95. doi: 10.18332/tid/128497 (PMC7676305; doi:10.18332/tid/128497)
Supplement: Supplementary file 1 [file TID-18-95-s1.pdf]

Figure 1. Flow chart of articles identification

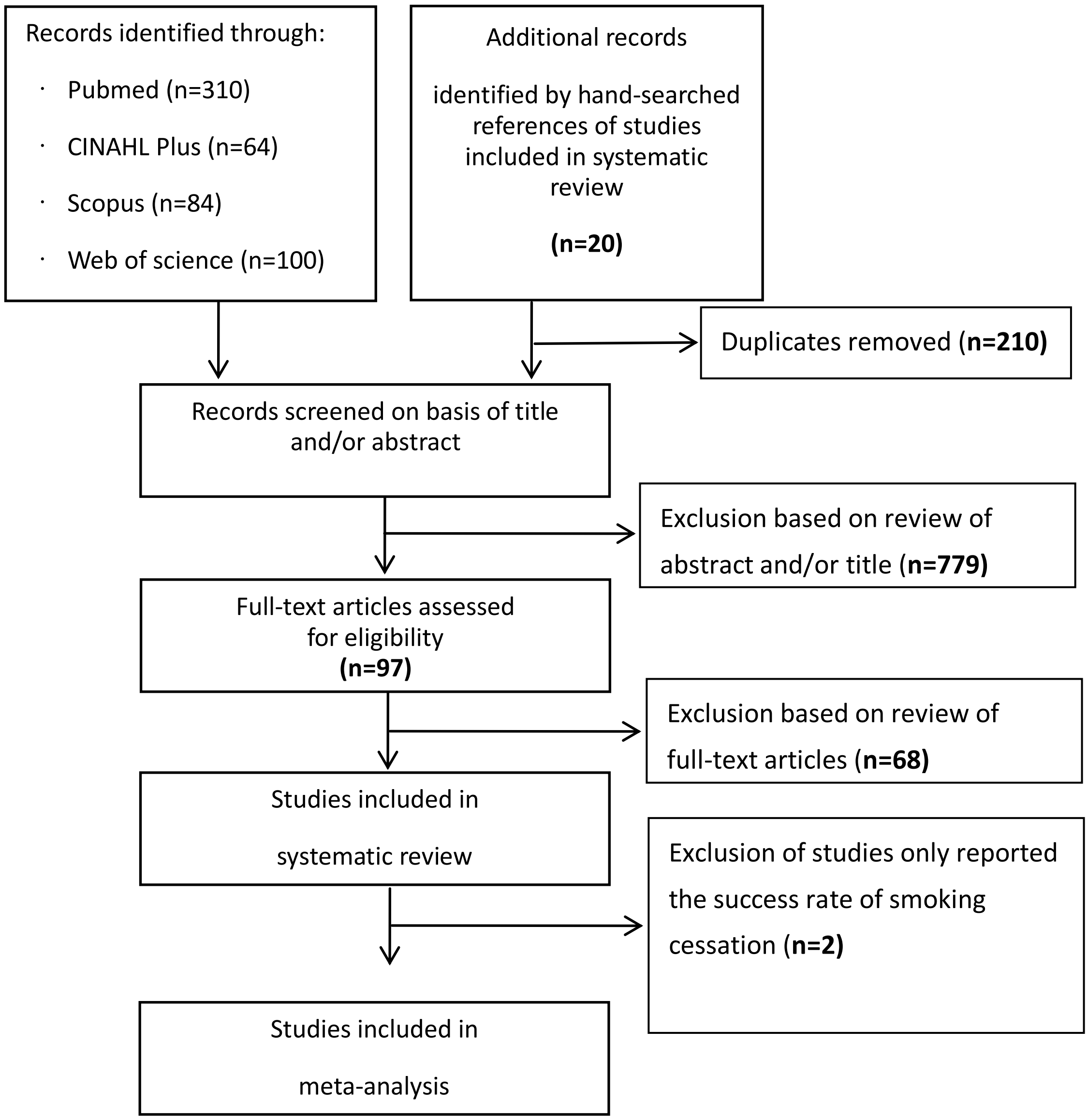

Note: The search terms were: [‘factors’ or ‘predictors’] and [‘smoking cessation’ or ‘smoking cessation interventions’ or ‘quit smoking’ or ‘stop smoking’]. The terms of [‘Japan’ or ‘Japanese’] were further limited when the papers were searched in four English electronic databases. We also searched the reference lists of publications included in the systematic review.

Table 1. Overall summary of studies reported success of quitting smoking in the Japanese Smoking Cessation Treatment program

First Author, Year	Language	Territory	No. of subjects	The success rate of smoking cessation		Factors associated with success of smoking cessation[a]	
				12 weeks	1 year	12 weeks	1 year
Uchida, 2007	Japanese	Kansai	116	61.2%	NA[b]	Gender, Tobacco Dependence (TDS), number of cigarettes per day, cohabitant with smokers.	NA
Yamamoto, 2007	Japanese	Tohoku	1627	54.0%	NA	NA	NA
Kurioka, 2008; 2009	Japanese	Kansai	61	47.5%	36.1%	Number of cigarettes per day, number of sessions participating to smoking cessation treatment (SCT)	Cohabitant with smokers
Ito, 2008	Japanese	Kyushu	105	84.8%	46.7%	TDS	Gender, TDS, having present diseases
Hirata, 2009	Japanese	Kansai	496	57.1%	NA	NA	NA
Tanaka, 2010	Japanese	Kyushu	100	52.0%	NA	TDS, completion of SCT program	NA
Shinoda, 2011	Japanese	Chubu	199	NA	NA	NA	
Sawayama,2011	Japanese	Chugoku	66	71.2%	48.5%	Age, age at smoking initiation, Subjective symptoms.	NA
Taniguchi, 2011	Japanese	Chubu	372	45.2%	NA	Gender, self-efficacy, varenicline	NA
Sato, 2011	Japanese	Tohoku	201	51.2%	NA	NA	NA
Satoh, 2012	Japanese	Tohoku	276	67.6%	17.8%	Gender	Gender
Miyagi, 2012	Japanese	Kanto	87	79.3%	64.4%	Number of sessions participating to SCT.	Number of sessions participating to SCT, Health counseling, Anxiety.

Okazaki, 2013	Japanese	Kyushu	230	72.2%	NA	Age, gender, having present diseases, having mental diseases, number of cigarettes per day.	NA
Yoshii, 2013	Japanese	Kyushu	133	66.2%	NA	Gender, side effects of varenicline, having mental diseases	NA
Nobata, 2013	Japanese	Chubu	133	65.4%	30.1%	Gender,weight, duration of SCT, age at smoking initiation.	NA
Taniguchi, 2013	English	Chubu	283	82.7%	68.6%	NA	NA
Iwaoka, 2014	English	Kanto	86	80.2%	NA	NA	NA
Yamazaki, 2015	Japanese	Hokkaido	125	60.8%	NA	NA	NA
Yasuda, 2015	Japanese	Kanto	130	71.5%	NA	Brinkman index, subjective symptoms, completion of SCT program.	NA
Iwaoka, 2016	English	Kanto	81	76.5%	NA	NA	NA
Shimadu, 2016	English	Chubu	193	46.6%	36.8%	NA	NA
Itakura, 2016	Japanese	Kanto	81	54.3%	NA	Motivation to quit smoking, number of sessions participating to SCT, self-efficacy.	
Ishii, 2017	Japanese	Kanto	190	70.5%	NA	**Male:** height, weight, self-efficacy, hypertension.	NA
Taniguchi,2017	English	Chubu	1320	41.0%	19.0%	Age, gender, FTND, desire to smoke, age at smoking initiation, previous abstinence, motivation to quit smoking, self-efficacy.	Desire to smoke.
Taniguchi H, 2018	Japanese	Kyushu	110	41.8%	25.5%	NA	Social nicotine dependence, self-efficacy, number of cigarettes per day.

Taniguchi C, 2018	English	Chubu	488	81.6%	NA	Self-efficacy	NA
Sugiyama,2018	Japanese	Kansai	230	68.3%	NA	Age, Brinkman index, number of sessions participating to SCT, completion of SCT program, having mental diseases, side effects of varenicline, decrement or discontinuation of varenicline	NA
Tomioka, 2019	English	Kansai	813	66.5%	NA	Age, number of cigarettes per day, duration of smoking years, exhaled CO concentration, having mental diseases.	NA

[a] factors are statistically associated with smoking cessation reported in the papers.

[b] Not available.

Definition of smoking status

In this review, success of smoking cessation at 12 weeks is defined as smoking abstinence at the end of SCT program (12 weeks) by clinical diagnosis or self-reported which is referred in the included studies. Maintained cessation at 1 year is defined as smoking abstinence by self-reported which is also referred in the included studies. In addition, to facilitate the comparison of the success rate among different studies at 12 weeks and/or 1 year, we recalculate the success rate of smoking cessation based on the extracted accurate data.

Figure 2. Forest plot of gender at 12 weeks

	Male		Female			Odds Ratio
Study or Subgroup	Events	Total	Events	Total	Weight	M-H, Fixed, 95% CI
Hirata et al., 2009	172	328	54	168	7.0%	2.33 [1.58, 3.44]
Ishii et al., 2017	95	127	39	63	2.7%	1.83 [0.96, 3.49]
Itakura et al., 2016	33	61	11	20	1.6%	0.96 [0.35, 2.66]
Ito et al., 2008	68	80	21	25	1.0%	1.08 [0.31, 3.70]
Iwaoka et al., 2014	41	53	28	33	1.6%	0.61 [0.19, 1.92]
Iwaoka et al., 2016	35	48	27	33	1.8%	0.60 [0.20, 1.78]
Kurioka et al., 2008	17	35	12	26	1.5%	1.10 [0.40, 3.05]
Miyagi et al., 2012	45	52	24	35	0.8%	2.95 [1.01, 8.58]
Nobata et al., 2013	78	113	9	20	1.0%	2.72 [1.04, 7.16]
Okazaki et al., 2013	127	167	39	63	2.8%	1.95 [1.05, 3.63]
Sato et al., 2011	69	119	34	82	3.5%	1.95 [1.10, 3.45]
Satoh et al., 2012	114	152	28	58	2.1%	3.21 [1.71, 6.05]
Sawayama et al., 2011	40	52	7	14	0.5%	3.33 [0.97, 11.41]
Sugiyama, 2018	113	160	44	70	3.7%	1.42 [0.79, 2.57]
Tanaka et al., 2010	43	78	11	22	1.6%	1.23 [0.48, 3.17]
Taniguchi et al., 2011	119	243	49	129	6.7%	1.57 [1.01, 2.42]
Taniguchi et al., 2017	399	921	142	399	23.0%	1.38 [1.09, 1.76]
Tomioka et al., 2019	195	284	93	149	7.8%	1.32 [0.87, 2.00]
Uchida，2007	49	72	22	44	1.8%	2.13 [0.99, 4.61]
Yamamoto, 2007	688	1181	192	446	23.8%	1.85 [1.48, 2.30]
Yasuda et al., 2015	67	97	26	33	2.5%	0.60 [0.24, 1.54]
Yoshii et al., 2013	68	92	20	41	1.5%	2.98 [1.38, 6.42]
**Total (95% CI)**		**4515**		**1973**	**100.0%**	**1.66 [1.49, 1.86]**
Total events	2675		932			

Heterogeneity: $Chi^2 = 31.61$, $df = 21$ ($P = 0.06$); $I^2 = 34\%$

Test for overall effect: $Z = 9.05$ ($P < 0.00001$)

Odds Ratio

M-H, Fixed, 95% CI

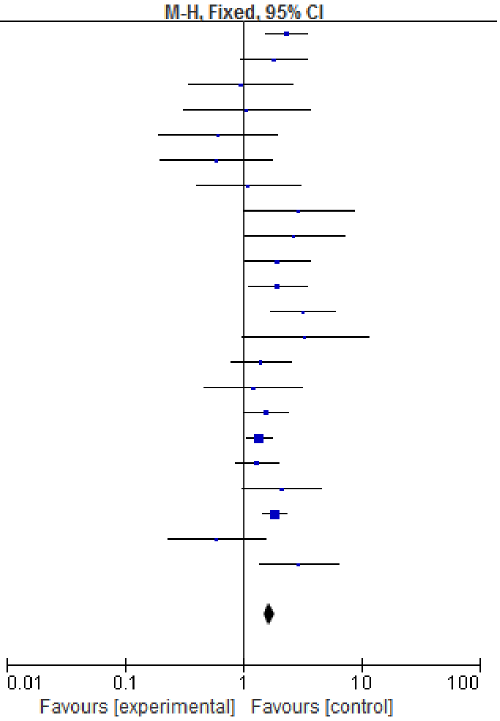

Figure 3. Funnel plot of gender at 12 weeks

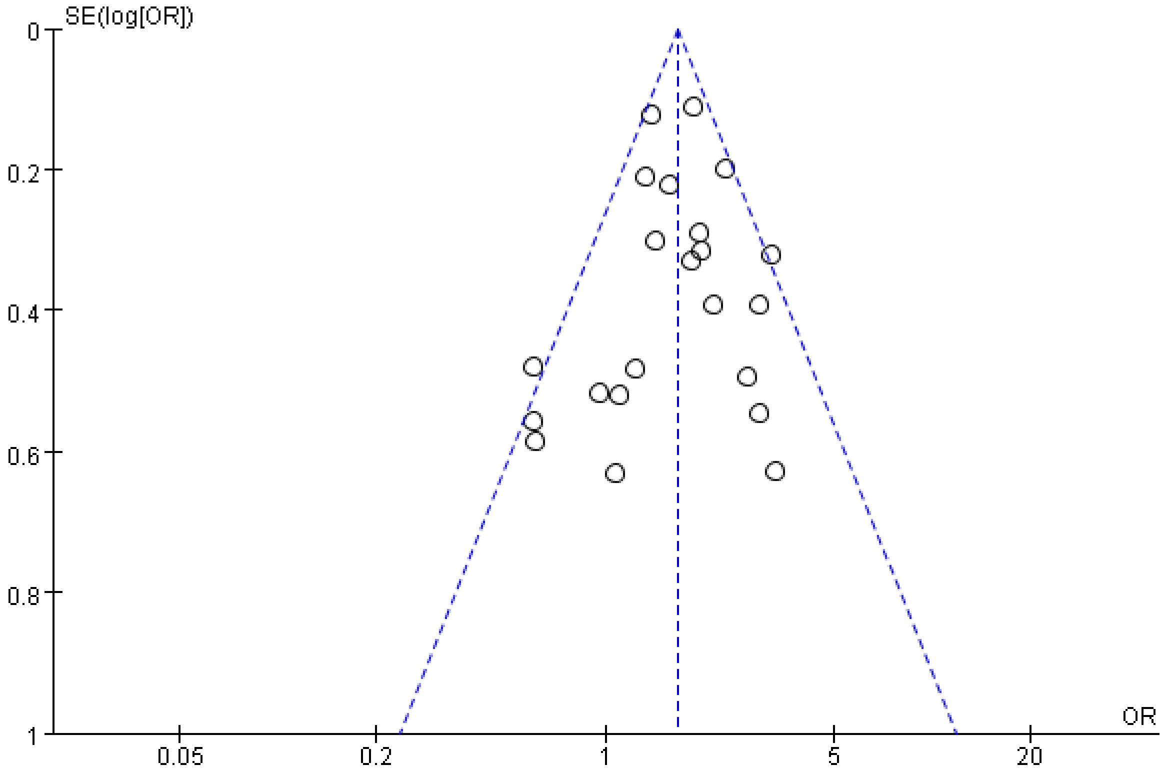

Figure 4. Forest plot of gender at 1 year

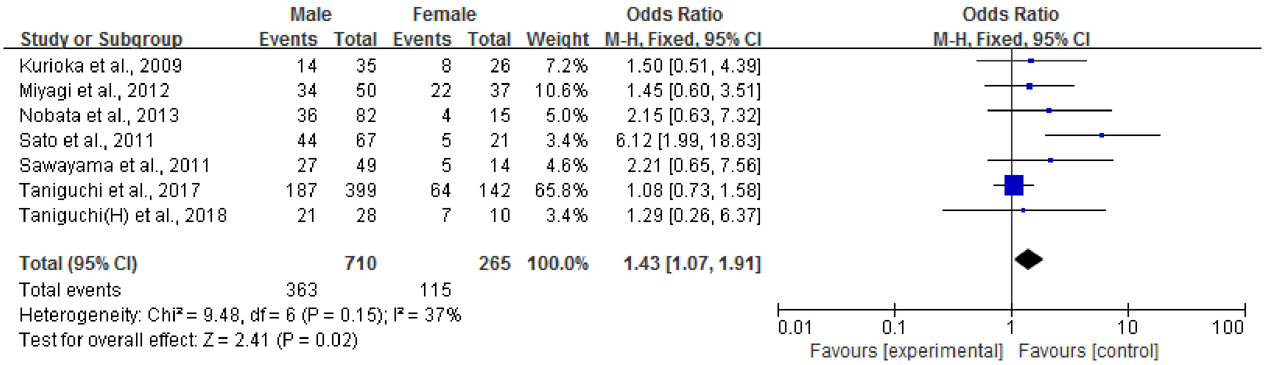

Figure 5. Funnel plot of gender at 1 year

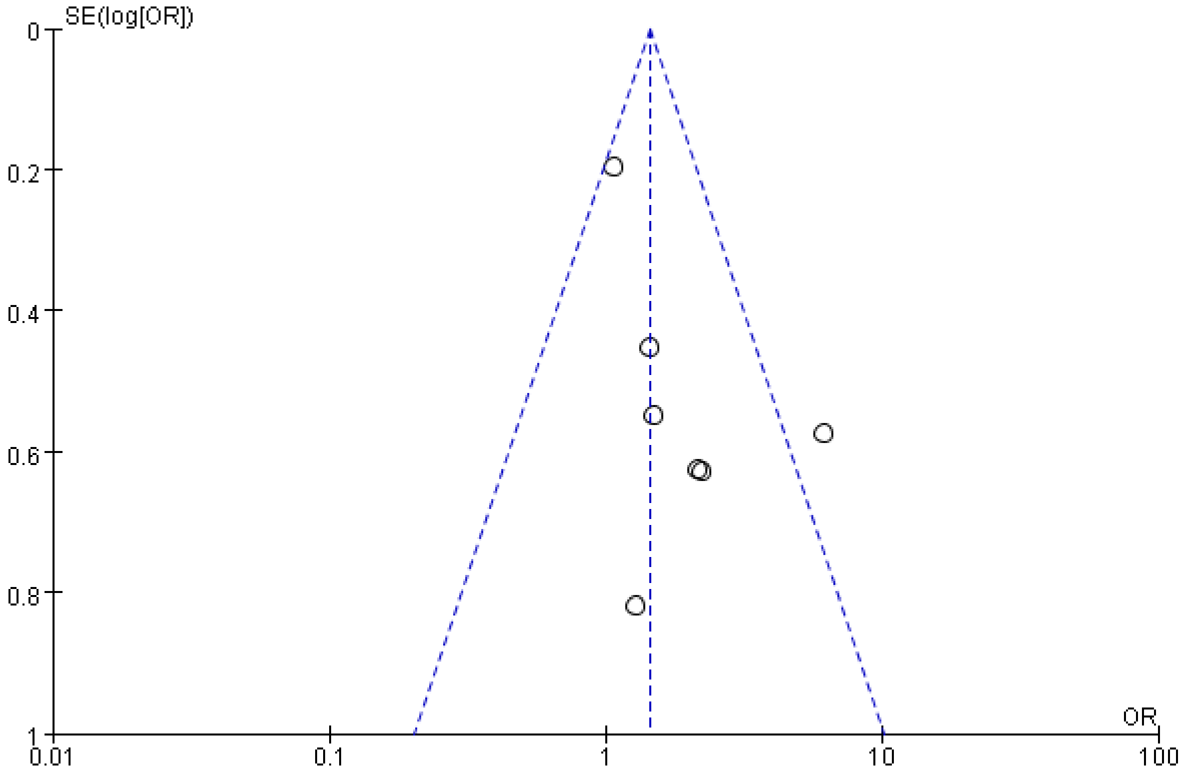

Figure 6. Forest plot of age at 12 weeks

	Success group			Failure group				Mean Difference
Study or Subgroup	Mean	SD	Total	Mean	SD	Total	Weight	IV, Random, 95% CI
Itakura et al., 2016	44.8	9	44	43.6	9.3	37	8.0%	1.20 [-2.81, 5.21]
Iwaoka et al., 2014	60.2	11.3	69	60.4	11.7	17	4.0%	-0.20 [-6.37, 5.97]
Iwaoka et al., 2016	60.2	11.7	62	59.8	13	19	3.6%	0.40 [-6.13, 6.93]
Miyagi et al., 2012	53.9	13.6	69	57.9	10.3	18	4.5%	-4.00 [-9.74, 1.74]
Nobata et al., 2013	51.8	12	78	51.2	12	34	6.0%	0.60 [-4.23, 5.43]
Sugiyama, 2018	59.2	15.1	157	51.7	18.6	73	5.9%	7.50 [2.62, 12.38]
Tanaka et al., 2010	56.2	1.9	52	51.9	1.8	48	26.8%	4.30 [3.57, 5.03]
Taniguchi et al., 2017	55.8	13.6	541	52.3	14.3	779	21.1%	3.50 [1.98, 5.02]
Tomioka et al., 2019	63.2	11.9	288	58.7	12.3	145	14.8%	4.50 [2.07, 6.93]
Yoshii et al., 2013	55.5	12.4	88	51.5	15.5	45	5.3%	4.00 [-1.22, 9.22]
**Total (95% CI)**			**1448**			**1215**	**100.0%**	**3.17 [1.84, 4.50]**

Heterogeneity: Tau² = 1.58; Chi² = 17.36, df = 9 (P = 0.04); I² = 48%
Test for overall effect: Z = 4.67 (P < 0.00001)

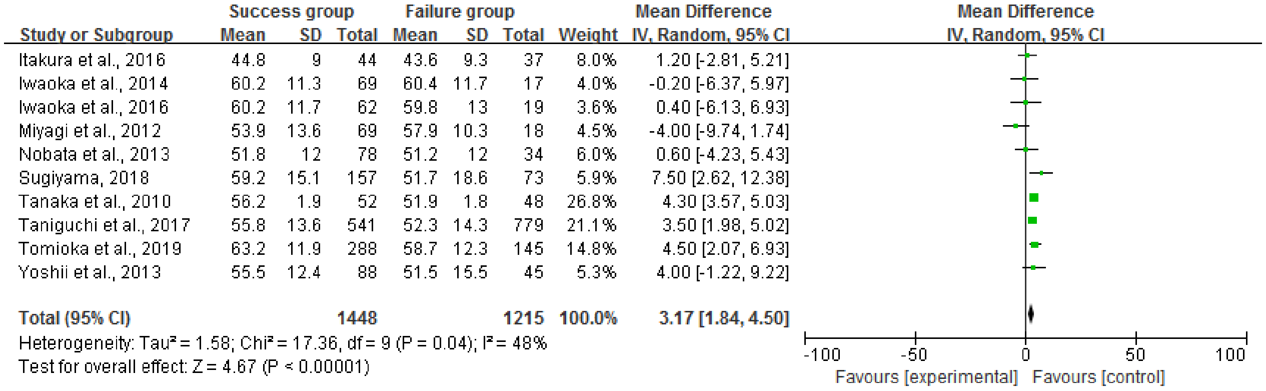

Figure 7. Funnel plot of age at 12 weeks

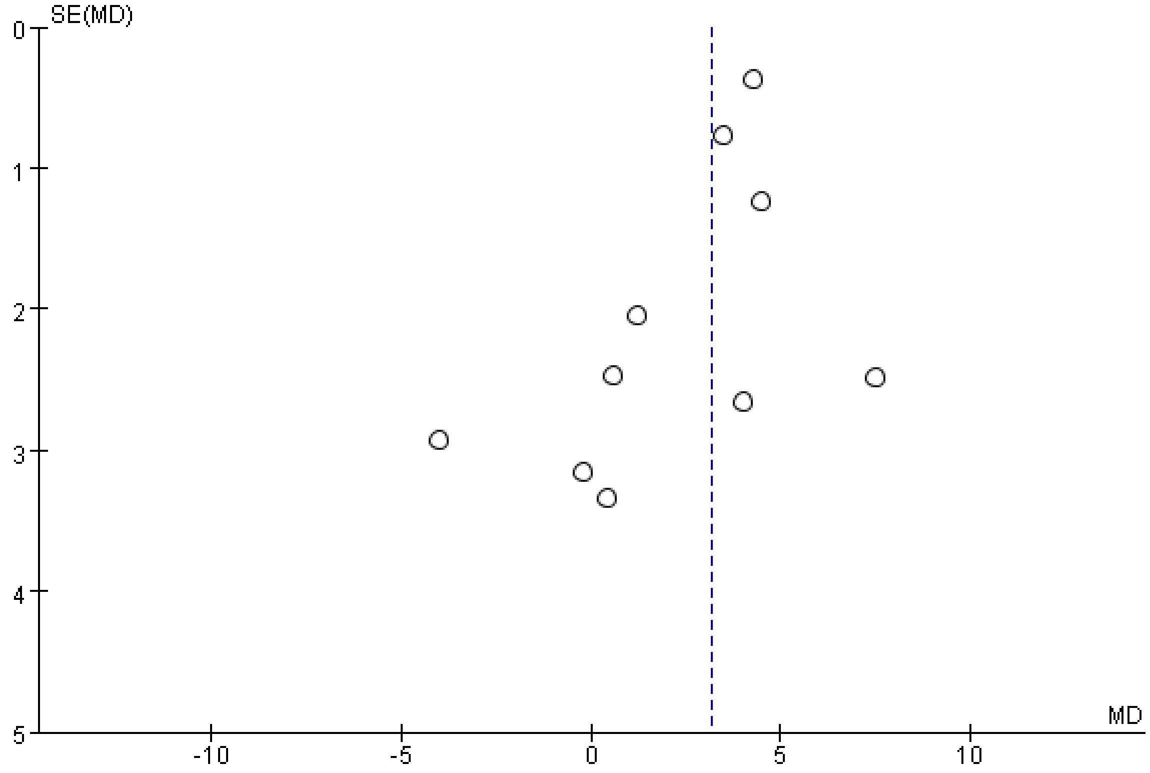

Figure 8. Forest plot of age at 12 weeks (≧50 years)

	≧50 years old		<50 years old			Odds Ratio
Study or Subgroup	Events	Total	Events	Total	Weight	M-H, Fixed, 95% CI
Okazaki et al., 2013	61	79	105	151	17.2%	1.48 [0.79, 2.79]
Sato et al., 2011	66	114	37	87	18.5%	1.86 [1.06, 3.27]
Taniguchi et al., 2011	106	210	62	162	36.2%	1.64 [1.08, 2.49]
Uchida, 2007	26	65	45	122	19.6%	1.14 [0.62, 2.12]
Yasuda et al., 2015	27	35	66	95	8.5%	1.48 [0.60, 3.65]
**Total (95% CI)**		**503**		**617**	**100.0%**	**1.54 [1.20, 1.99]**
Total events	286		315			

Heterogeneity: Chi² = 1.45, df = 4 (P = 0.84); I² = 0%
Test for overall effect: Z = 3.33 (P = 0.0009)

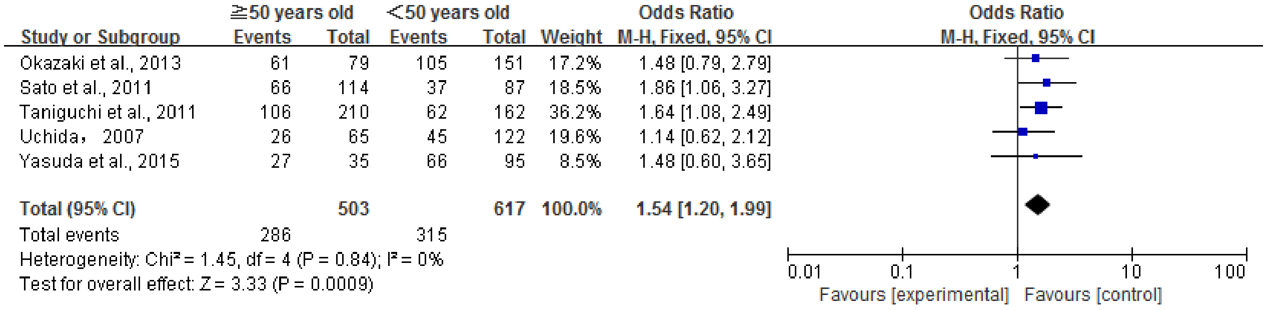

Figure 9. Funnel plot of age at 12 weeks (≧50 years)

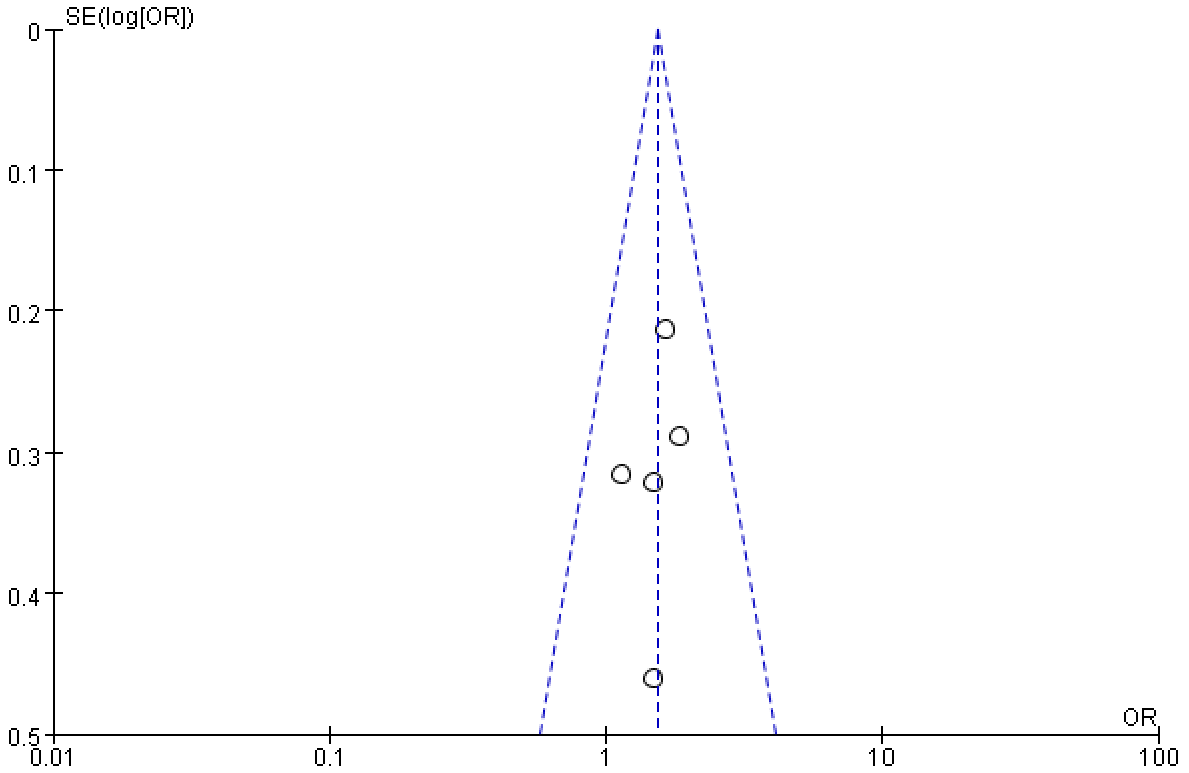

Figure 10. Forest plot of smoking cessation drug at 12 weeks

Study or Subgroup	Varenicline Events	Varenicline Total	Nicotine patch Events	Nicotine patch Total	Weight	Odds Ratio M-H, Fixed, 95% CI
Miyagi et al., 2012	48	60	21	27	4.1%	1.14 [0.38, 3.45]
Nobata et al., 2013	52	68	35	65	6.0%	2.79 [1.33, 5.86]
Shinoda et al., 2011	127	150	36	49	5.9%	1.99 [0.92, 4.32]
Taniguchi et al., 2011	150	309	17	56	10.5%	2.16 [1.17, 3.99]
Taniguchi et al., 2017	474	1090	67	230	44.3%	1.87 [1.37, 2.55]
Tomioka et al., 2019	141	202	147	231	29.3%	1.32 [0.88, 1.98]
**Total (95% CI)**		**1879**		**658**	**100.0%**	**1.77 [1.44, 2.18]**
Total events	992		323			

Heterogeneity: $Chi^2 = 4.70$, df = 5 (P = 0.45); $I^2 = 0\%$
Test for overall effect: Z = 5.48 (P < 0.00001)

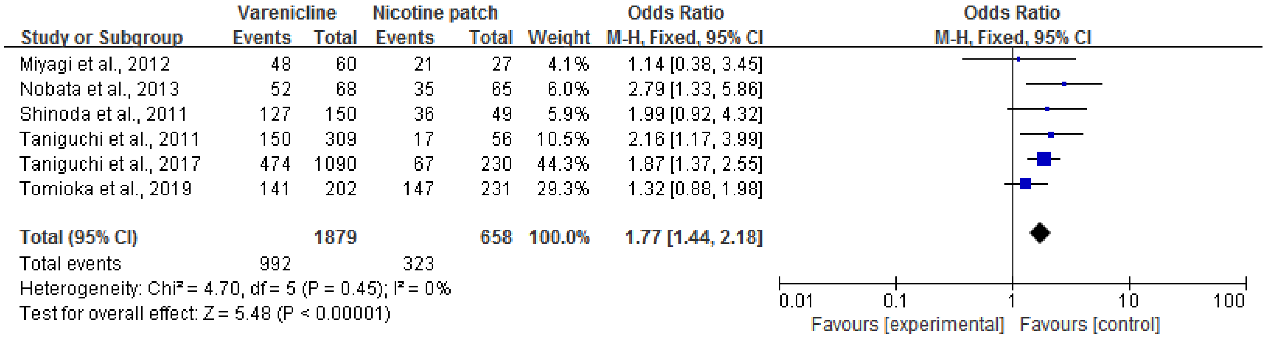

Figure 11. Funnel plot of smoking cessation drug at 12 weeks

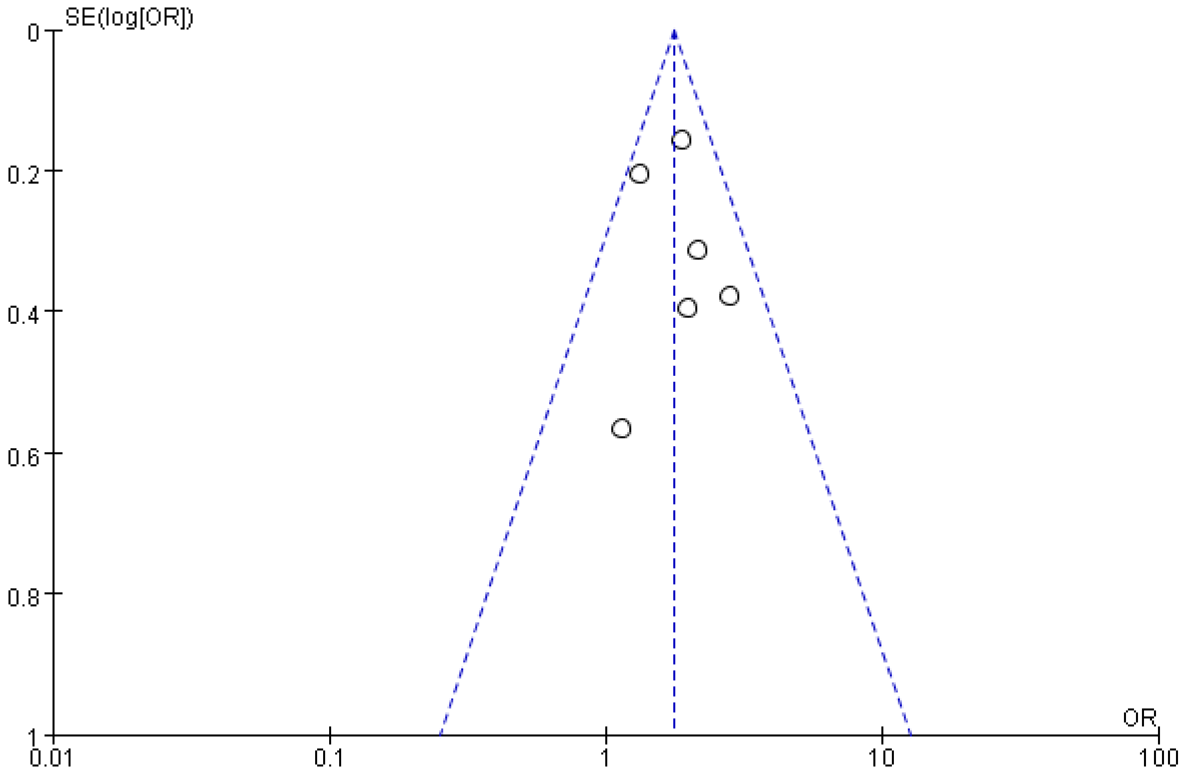

Figure 12. Forest plot of the completion of SCT program at 12 weeks

Study or Subgroup	Completed Events	Completed Total	Not completed Events	Not completed Total	Weight	Odds Ratio M-H, Random, 95% CI
Itakura et al., 2016	41	50	3	31	17.9%	42.52 [10.57, 171.08]
Ito et al., 2008	34	34	55	71	10.7%	20.51 [1.19, 353.01]
Sugiyama, 2018	114	131	157	230	21.7%	3.12 [1.75, 5.57]
Tanaka et al., 2010	52	68	0	32	10.7%	206.82 [12.00, 3565.78]
Uchida, 2007	11	16	60	100	19.3%	1.47 [0.47, 4.54]
Yasuda et al., 2015	69	74	24	56	19.7%	18.40 [6.43, 52.62]
**Total (95% CI)**		**373**		**520**	**100.0%**	**11.72 [3.24, 42.42]**
Total events	321		299			

Heterogeneity: $Tau^2 = 1.90$; $Chi^2 = 30.37$, df = 5 (P < 0.0001); $I^2 = 84\%$
Test for overall effect: Z = 3.75 (P = 0.0002)

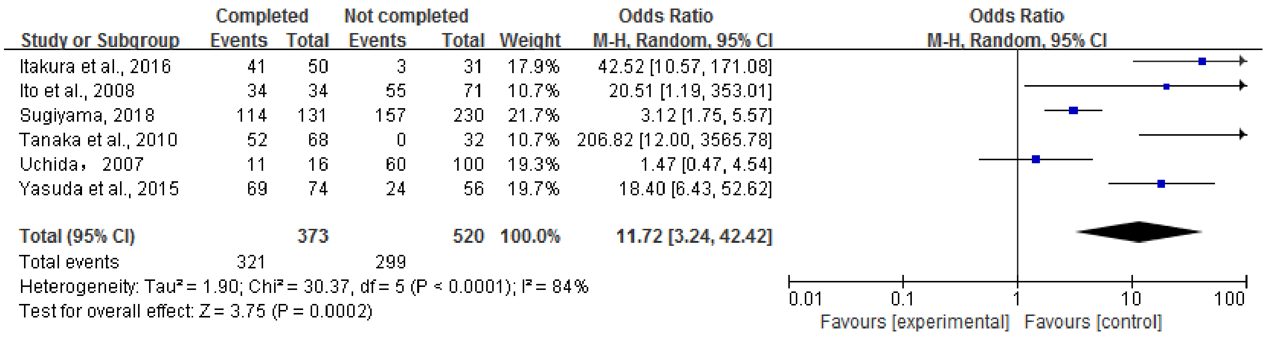

Figure 13. Funnel plot of the completion of SCT program at 12 weeks

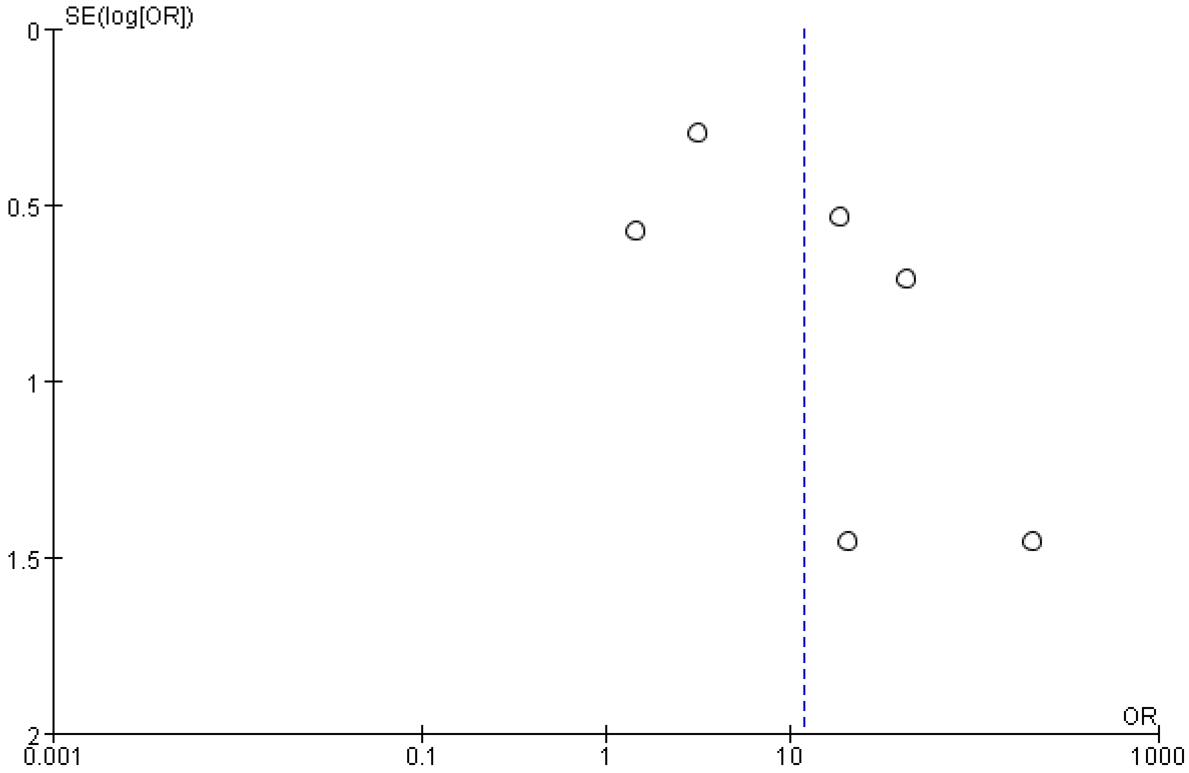

Figure 14. Forest plot of Brinkman index at 12 weeks

	Success group			Failure group				Mean Difference
Study or Subgroup	Mean	SD	Total	Mean	SD	Total	Weight	IV, Fixed, 95% CI
Iwaoka et al., 2014	865	512	69	834	485	17	2.6%	31.00 [-229.28, 291.28]
Iwaoka et al., 2016	799.6	410.8	62	872.5	495.3	19	3.0%	-72.90 [-317.96, 172.16]
Miyagi et al., 2012	878.7	452.6	69	870.3	442.4	18	3.3%	8.40 [-222.19, 238.99]
Nobata et al., 2013	767	419	78	793	433	34	5.9%	-26.00 [-198.71, 146.71]
Sugiyama, 2018	775.6	588	157	620.3	372.4	73	11.3%	155.30 [29.77, 280.83]
Tanaka et al., 2010	1,013	102	52	949	181	48	52.3%	64.00 [5.77, 122.23]
Tomioka et al., 2019	843.1	529.6	288	876	507.7	145	16.8%	-32.90 [-135.71, 69.91]
Yoshii et al., 2013	872.4	491.3	88	829.1	553.5	45	4.8%	43.30 [-148.25, 234.85]
**Total (95% CI)**			**863**			**399**	**100.0%**	**44.91 [2.80, 87.02]**

Heterogeneity: Chi² = 7.23, df = 7 (P = 0.41); I² = 3%
Test for overall effect: Z = 2.09 (P = 0.04)

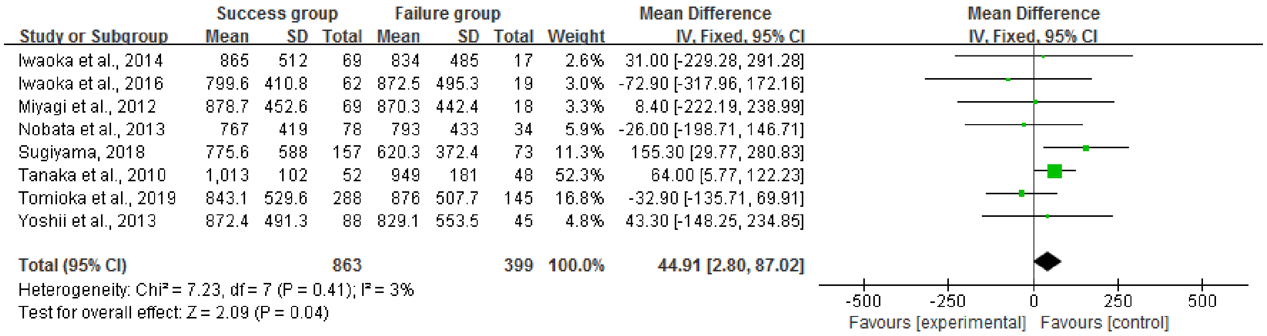

Figure 15. Funnel plot of Brinkman index at 12 weeks

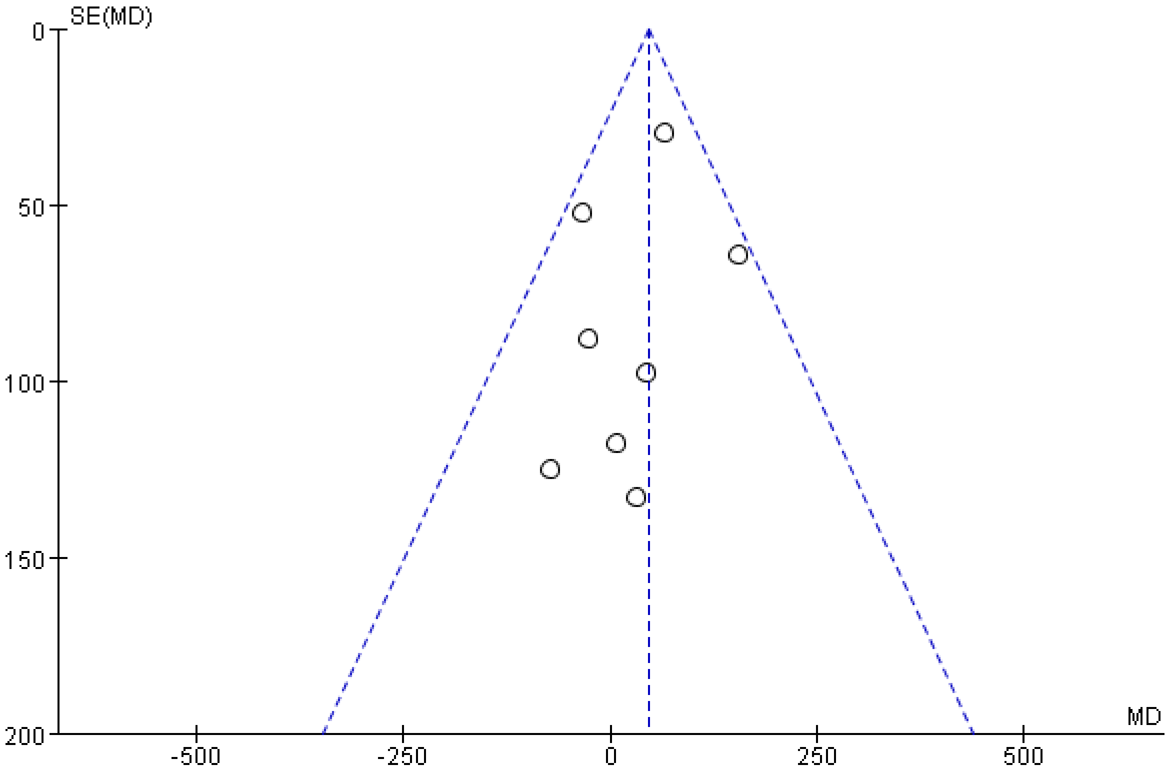

Figure 16. Forest plot of Nicotine dependence (TDS) at 12 weeks

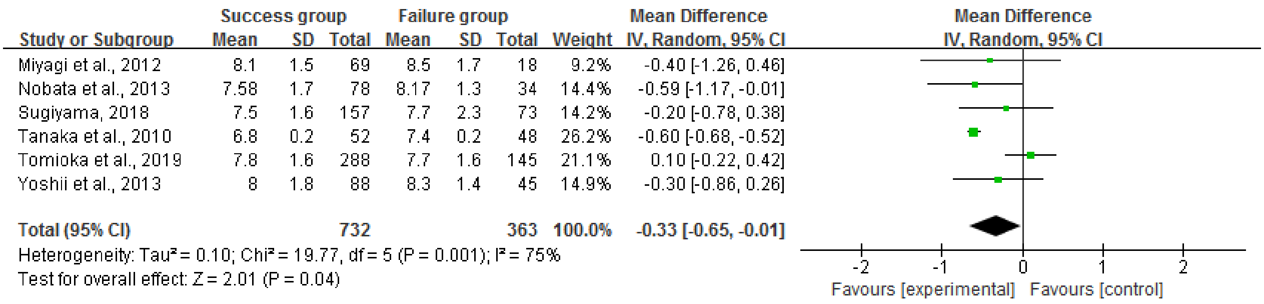

Figure 17. Funnel plot of Nicotine dependence (TDS) at 12 weeks

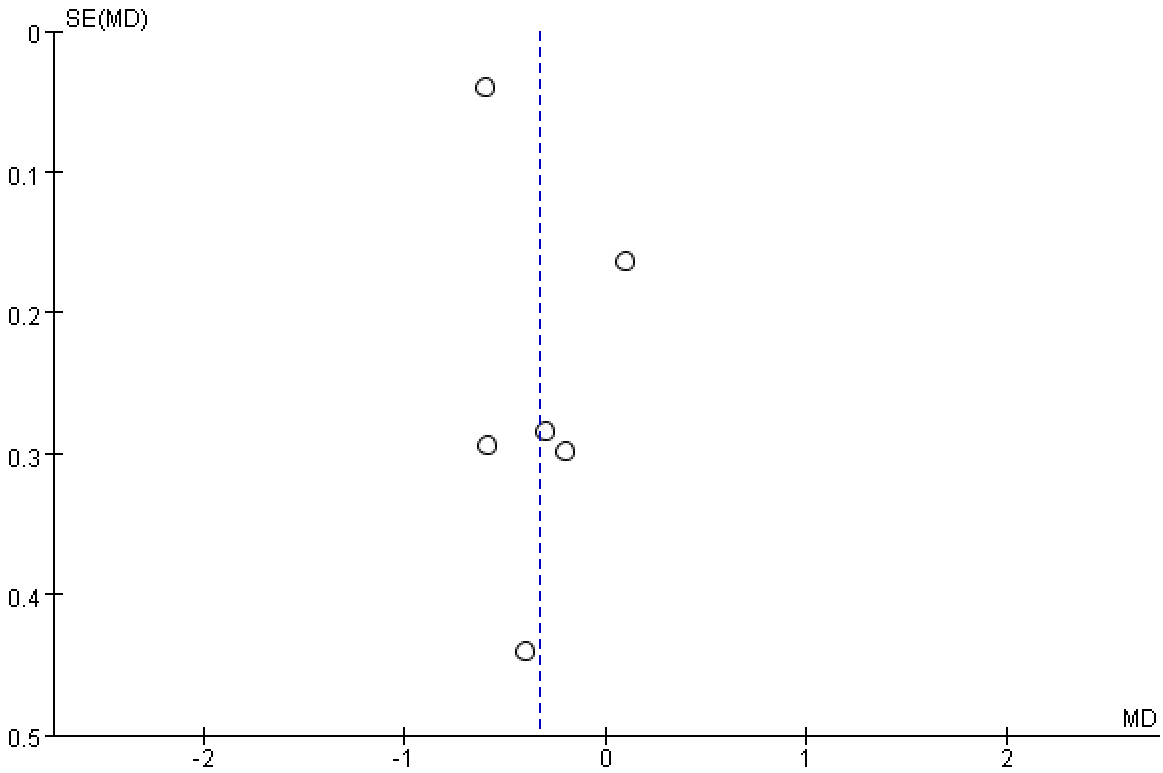

Figure 18. Forest plot of the number of cigarettes smoking per day at 12 weeks

Study or Subgroup	Success group Mean	Success group SD	Success group Total	Failure group Mean	Failure group SD	Failure group Total	Weight	Mean Difference IV, Fixed, 95% CI
Iwaoka et al., 2014	22.6	10.5	69	21.9	11.5	17	8.2%	0.70 [-5.30, 6.70]
Iwaoka et al., 2016	20.1	7.8	62	22.7	10.7	19	10.9%	-2.60 [-7.79, 2.59]
Miyagi et al., 2012	26	9.2	69	24.5	11.3	18	9.2%	1.50 [-4.15, 7.15]
Nobata et al., 2013	23.3	9.2	78	27.6	16	34	8.9%	-4.30 [-10.05, 1.45]
Sugiyama, 2018	20.7	14.5	157	21.6	20.3	73	11.0%	-0.90 [-6.08, 4.28]
Tomioka et al., 2019	20.7	10.5	288	24.3	12.6	145	51.8%	-3.60 [-5.98, -1.22]
**Total (95% CI)**			**723**			**306**	**100.0%**	**-2.44 [-4.15, -0.72]**

Heterogeneity: Chi² = 4.57, df = 5 (P = 0.47); I² = 0%
Test for overall effect: Z = 2.78 (P = 0.005)

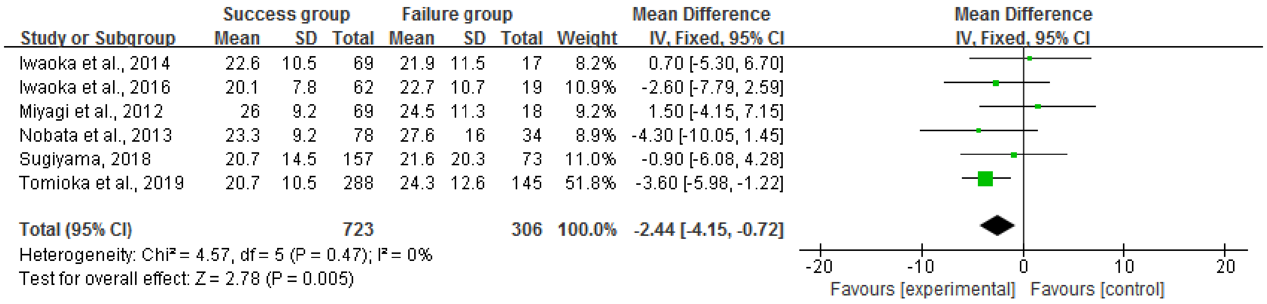

Figure 19. Funnel plot of the number of cigarettes smoking per day at 12 weeks

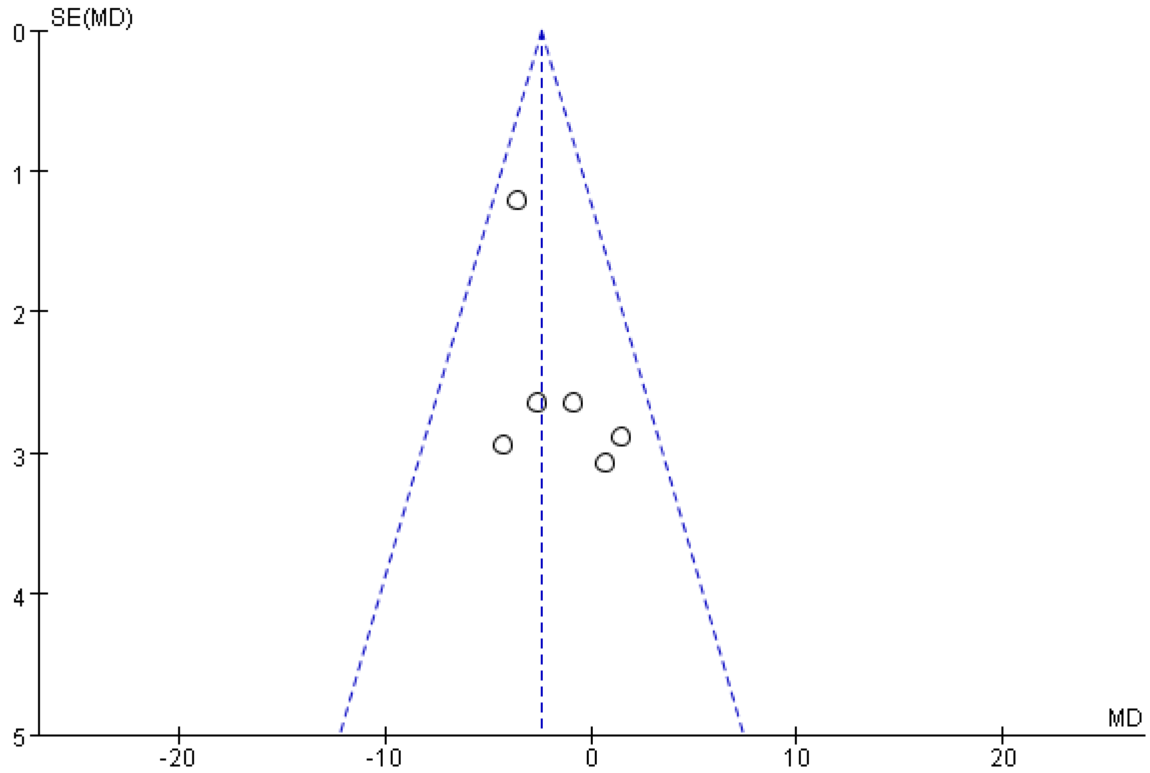

Figure 20. Forest plot of the number of smoking years at 12 weeks

	Success group			Failure group				Mean Difference
**Study or Subgroup**	**Mean**	**SD**	**Total**	**Mean**	**SD**	**Total**	**Weight**	**IV, Fixed, 95% CI**
Itakura et al., 2016	24.5	8.9	44	24.3	8.4	37	18.6%	0.20 [-3.57, 3.97]
Iwaoka et al., 2014	38.1	12.2	69	38.8	11.3	17	7.1%	-0.70 [-6.79, 5.39]
Iwaoka et al., 2016	38.9	11.3	62	38.4	12.1	19	7.1%	0.50 [-5.62, 6.62]
Miyagi et al., 2012	33.6	14	69	36.9	9.7	18	8.6%	-3.30 [-8.87, 2.27]
Nobata et al., 2013	32.3	10.7	78	33	10.8	34	14.1%	-0.70 [-5.04, 3.64]
Tomioka et al., 2019	40.7	12.3	288	36.7	12.2	145	44.5%	4.00 [1.56, 6.44]
**Total (95% CI)**			**610**			**270**	**100.0%**	**1.42 [-0.21, 3.05]**

Heterogeneity: Chi² = 8.92, df = 5 (P = 0.11); I² = 44%
Test for overall effect: Z = 1.71 (P = 0.09)

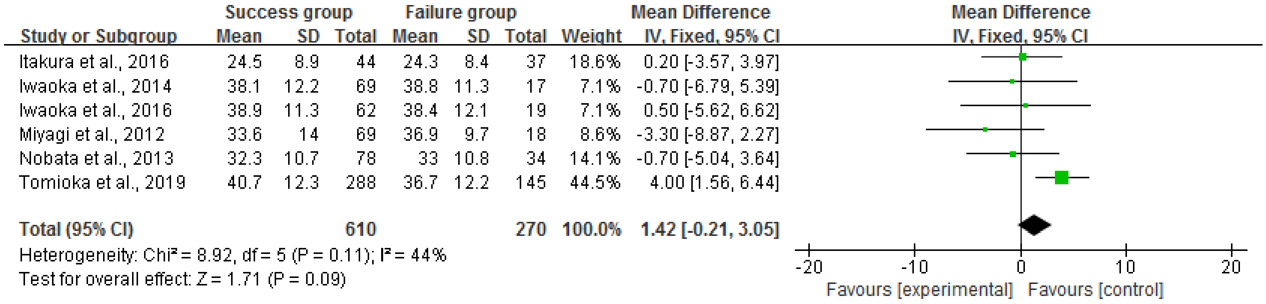

Figure 21. Funnel plot of the number of smoking years at 12 weeks

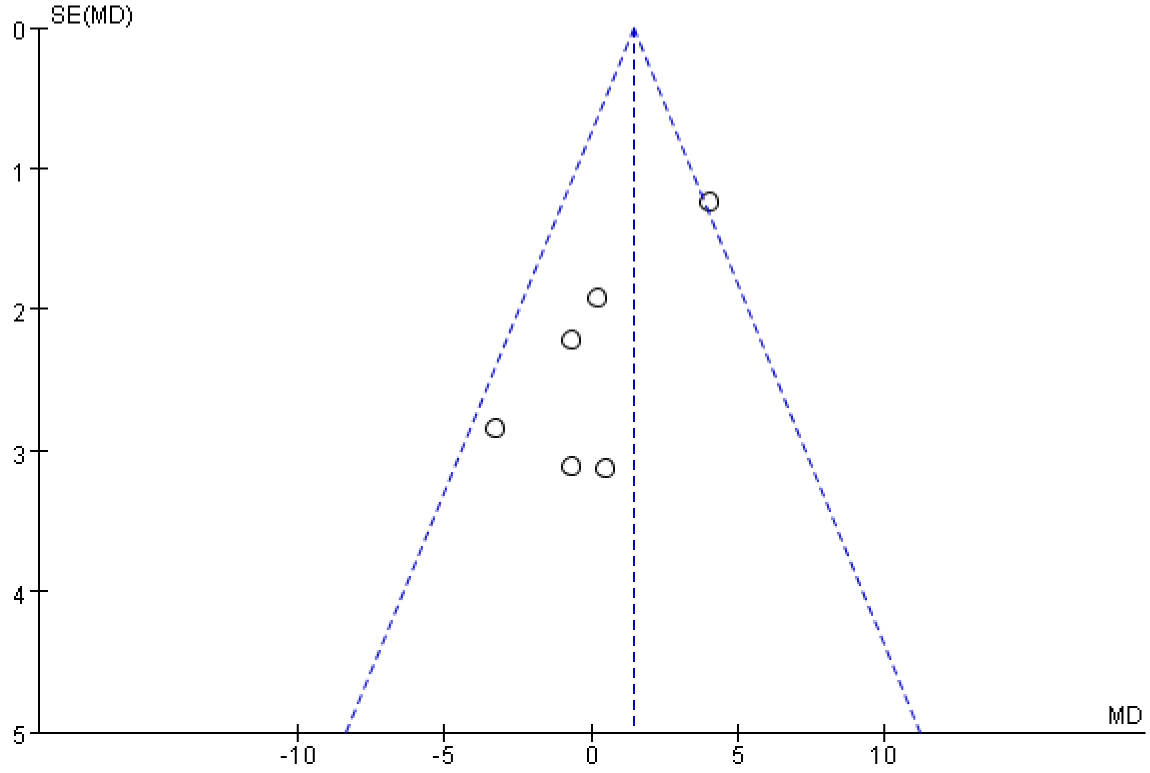

Figure 22. Forest plot of previous abstinence at 12 weeks

Study or Subgroup	Not experienced Events	Not experienced Total	Experienced Events	Experienced Total	Weight	Odds Ratio M-H, Fixed, 95% CI
Ishii et al., 2017	58	77	74	111	7.1%	1.53 [0.80, 2.93]
Itakura et al., 2016	16	30	28	51	4.6%	0.94 [0.38, 2.32]
Sawayama et al., 2011	26	37	21	29	3.3%	0.90 [0.31, 2.64]
Sugiyama, 2018	50	78	102	145	12.1%	0.75 [0.42, 1.35]
Taniguchi et al., 2017	114	326	419	975	64.4%	0.71 [0.55, 0.93]
Yasuda et al., 2015	46	71	47	59	8.5%	0.47 [0.21, 1.04]
**Total (95% CI)**		**619**		**1370**	**100.0%**	**0.77 [0.63, 0.95]**
Total events	310		691			

Heterogeneity: Chi² = 6.31, df = 5 (P = 0.28); I² = 21%
Test for overall effect: Z = 2.50 (P = 0.01)

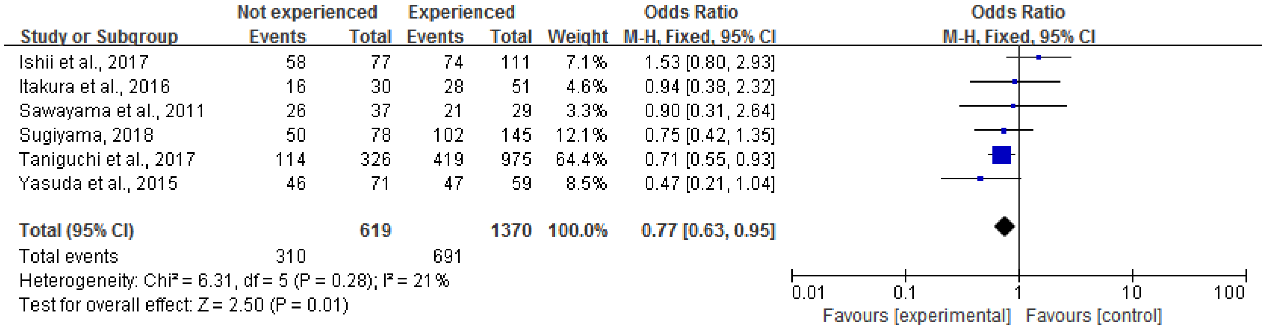

Figure 23. Funnel plot of previous abstinence at 12 weeks

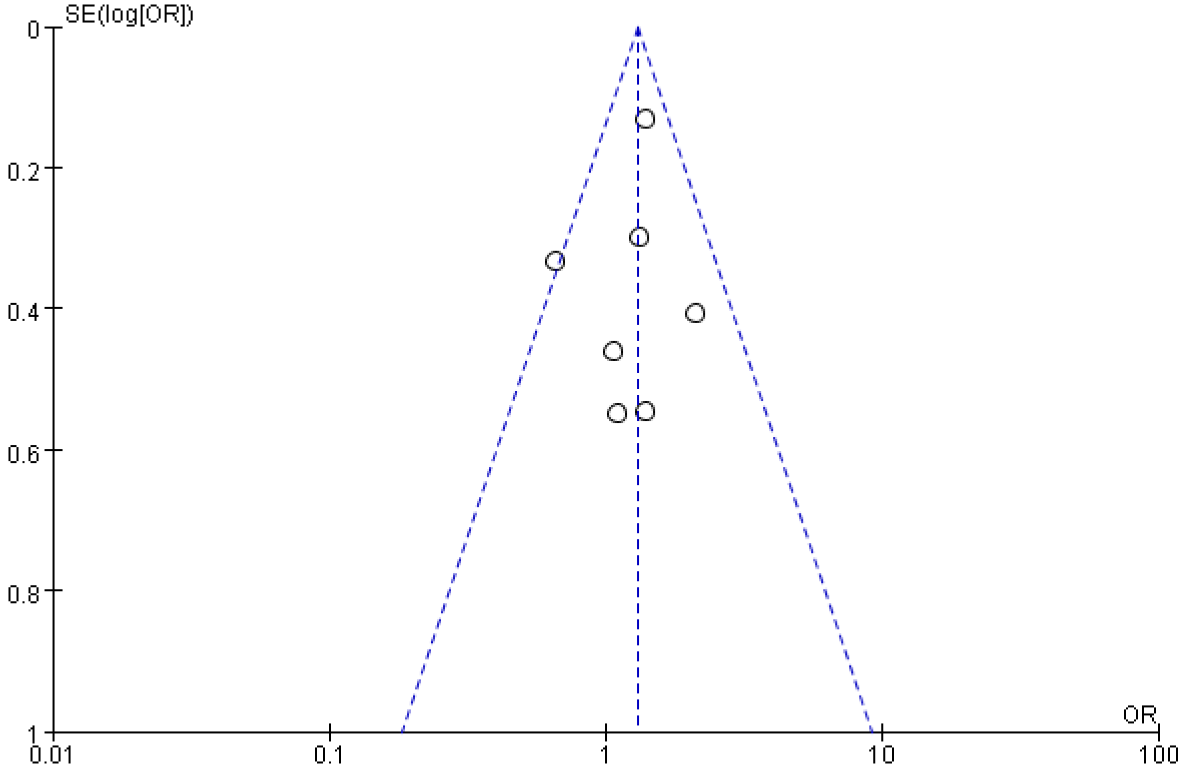

Figure 24. Forest plot of having present diseases at 12 weeks

Study or Subgroup	Absence Events	Absence Total	Presence Events	Presence Total	Weight	Odds Ratio M-H, Random, 95% CI
Ishii et al., 2017	24	37	110	153	13.9%	0.72 [0.34, 1.55]
Nobata et al., 2013	51	60	36	73	12.9%	5.82 [2.50, 13.55]
Okazaki et al., 2013	117	151	49	79	16.0%	2.11 [1.16, 3.81]
Sawayama et al., 2011	27	33	20	33	10.0%	2.92 [0.95, 9.03]
Sugiyama, 2018	37	49	116	176	14.4%	1.59 [0.77, 3.28]
Taniguchi et al., 2017	82	192	459	1128	19.3%	1.09 [0.80, 1.48]
Yasuda et al., 2015	55	80	38	50	13.4%	0.69 [0.31, 1.55]
**Total (95% CI)**		**602**		**1692**	**100.0%**	**1.56 [0.95, 2.55]**
Total events	393		828			

Heterogeneity: Tau² = 0.30; Chi² = 22.83, df = 6 (P = 0.0009); I² = 74%
Test for overall effect: Z = 1.76 (P = 0.08)

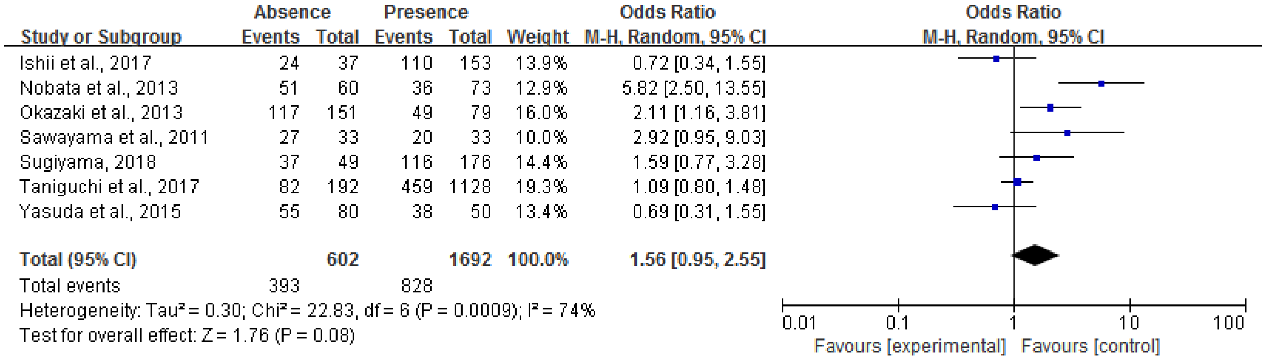

Figure 25. Funnel plot of having present diseases at 12 weeks

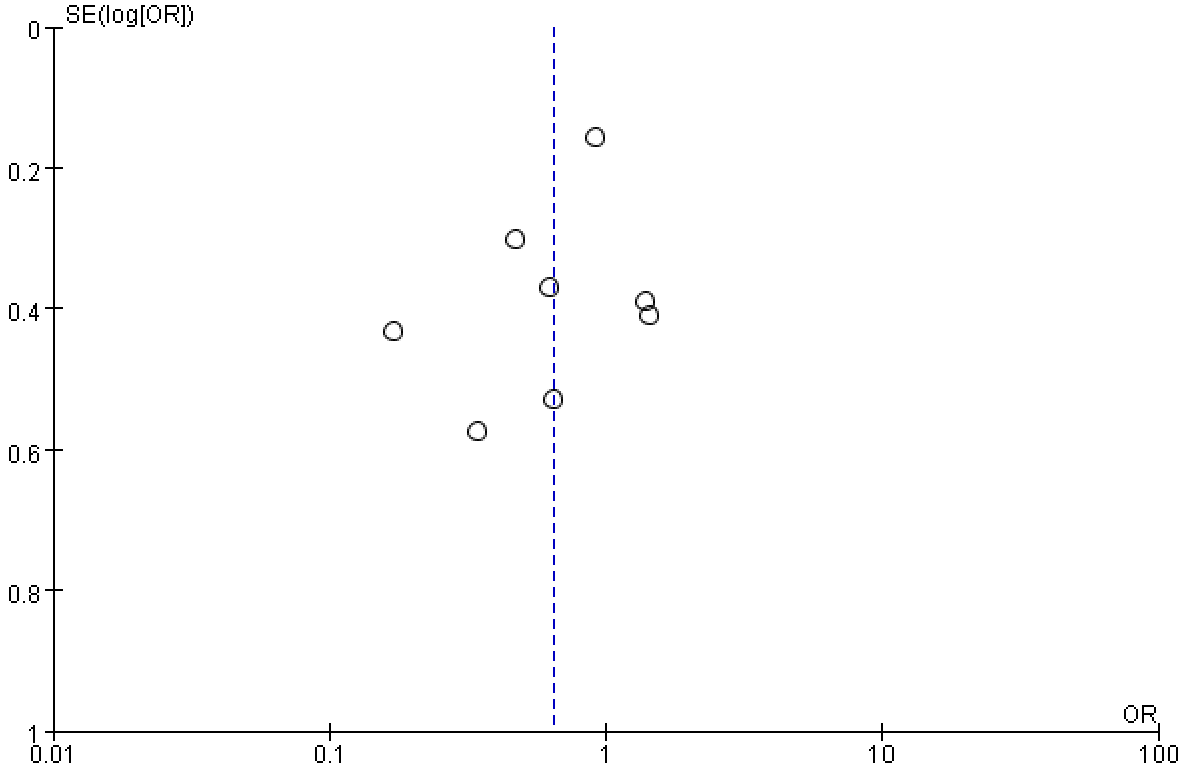

Figure 26. Forest plot of having mental diseases at 12 weeks

Study or Subgroup	Absence Events	Absence Total	Presence Events	Presence Total	Weight	Odds Ratio M-H, Fixed, 95% CI
Ishii et al., 2017	115	159	19	31	12.7%	1.65 [0.74, 3.68]
Nobata et al., 2013	82	117	5	16	3.8%	5.15 [1.67, 15.94]
Okazaki et al., 2013	162	214	4	16	2.6%	9.35 [2.89, 30.23]
Sugiyama, 2018	145	200	8	24	5.7%	5.27 [2.14, 13.02]
Taniguchi et al., 2011	140	286	28	86	31.8%	1.99 [1.20, 3.30]
Tomioka et al., 2019	223	309	65	124	37.3%	2.35 [1.53, 3.62]
Yoshii et al., 2013	80	115	8	18	6.1%	2.86 [1.04, 7.85]
**Total (95% CI)**		**1400**		**315**	**100.0%**	**2.63 [2.03, 3.41]**
Total events	947		137			

Heterogeneity: Chi² = 10.88, df = 6 (P = 0.09); I² = 45%

Test for overall effect: Z = 7.32 (P < 0.00001)

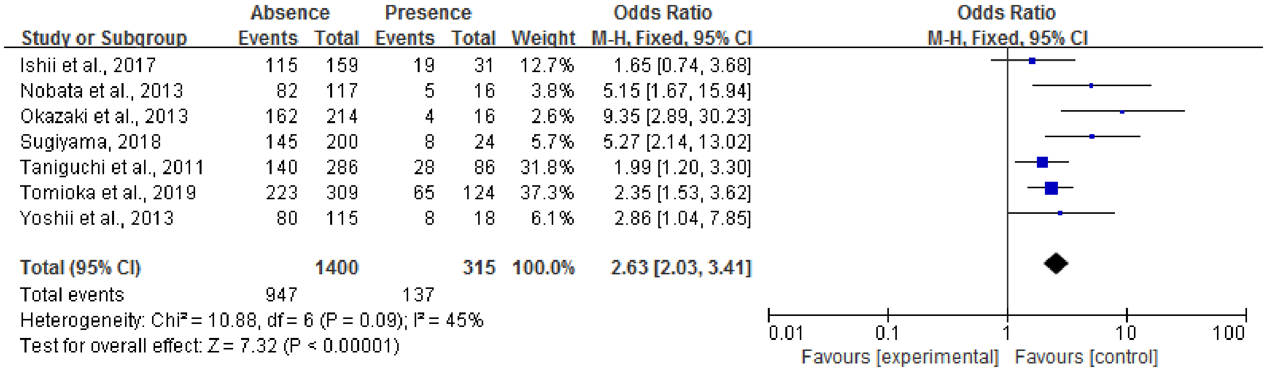

Figure 27. Funnel plot of having mental diseases at 12 weeks

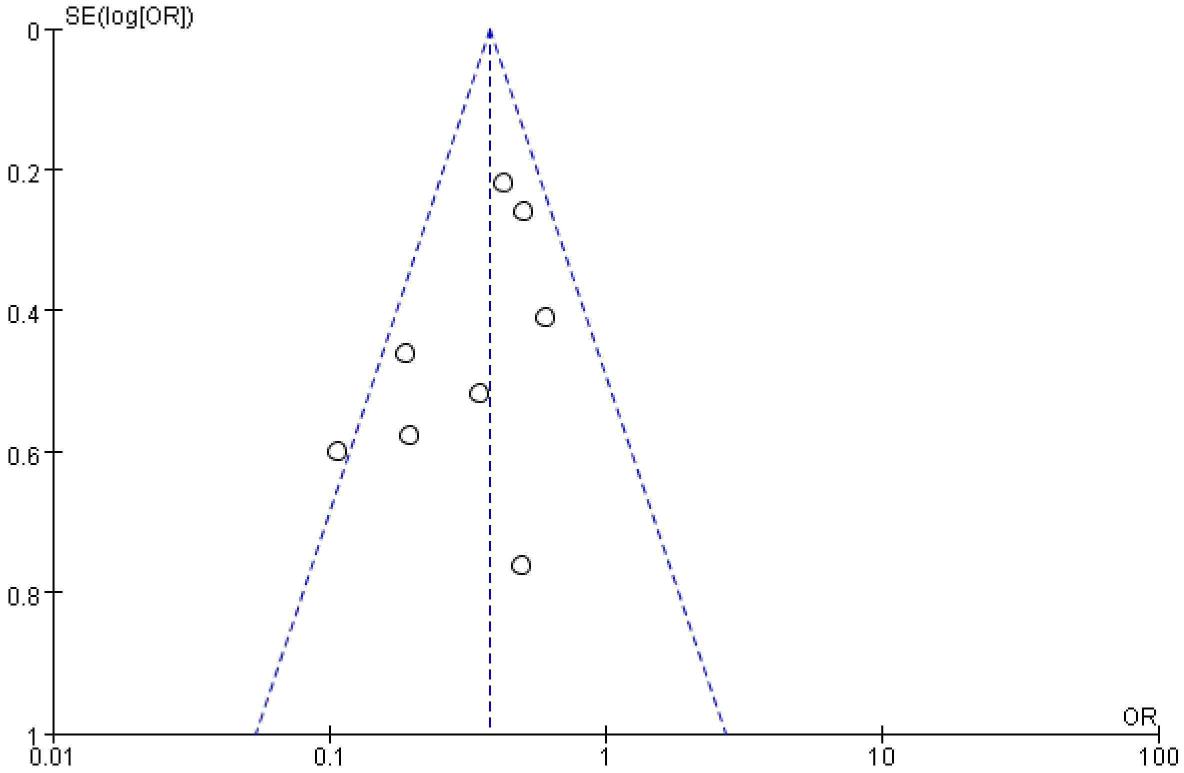

Figure 28. Forest plot of having respiratory diseases at 12 weeks

Study or Subgroup	Absence Events	Absence Total	Presence Events	Presence Total	Weight	Odds Ratio M-H, Fixed, 95% CI
Ishii et al., 2017	85	122	49	68	19.9%	0.89 [0.46, 1.72]
Iwaoka et al., 2016	42	51	20	30	4.6%	2.33 [0.82, 6.64]
Okazaki et al., 2013	162	223	4	7	2.2%	1.99 [0.43, 9.16]
Taniguchi et al., 2011	144	329	24	43	24.9%	0.62 [0.32, 1.17]
Tomioka et al., 2019	178	269	110	164	48.3%	0.96 [0.64, 1.45]
**Total (95% CI)**		**994**		**312**	**100.0%**	**0.95 [0.71, 1.26]**
Total events	611		207			

Heterogeneity: Chi² = 5.54, df = 4 (P = 0.24); I² = 28%
Test for overall effect: Z = 0.37 (P = 0.71)

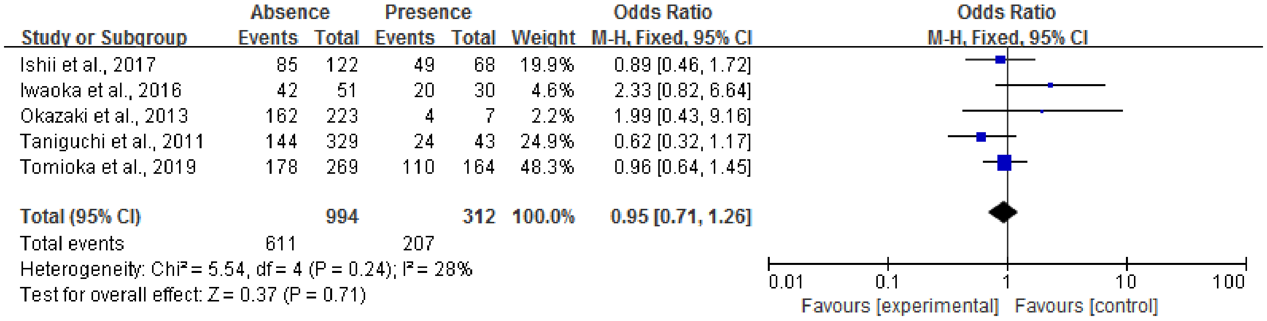

Figure 29. Funnel plot of having respiratory diseases at 12 weeks

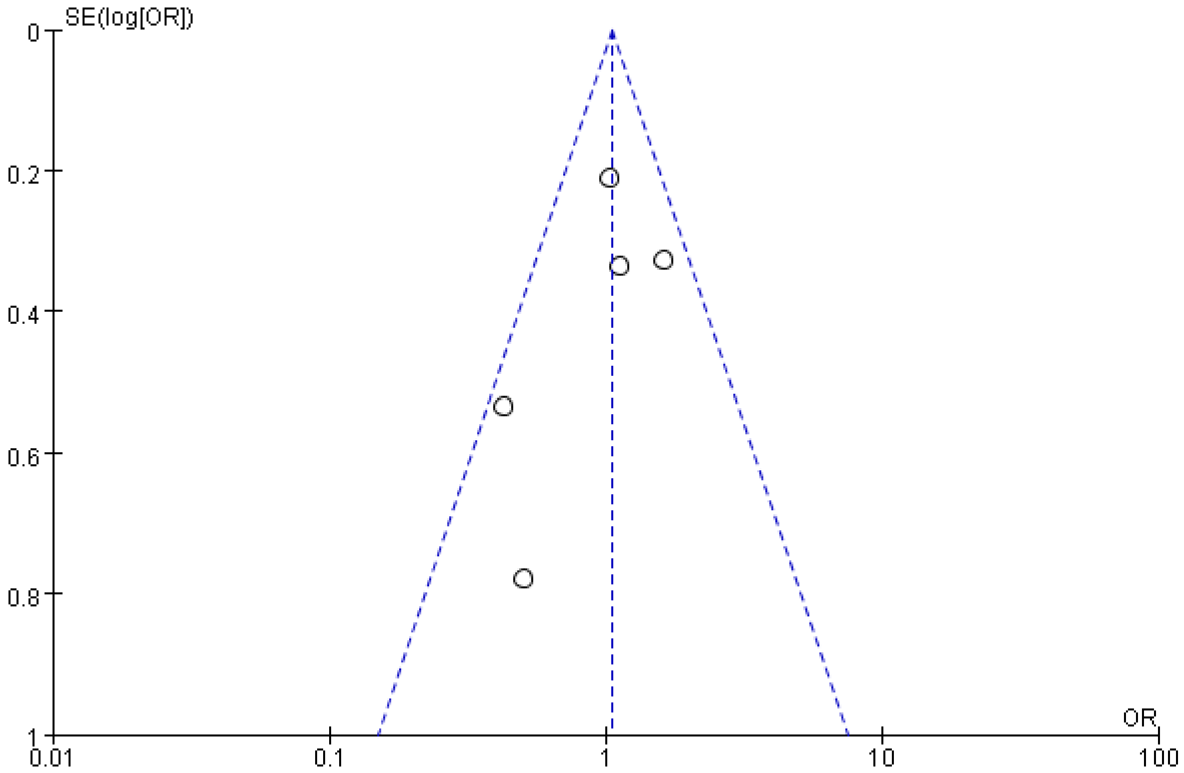

Figure 30. Forest plot of having diabetes at 12 weeks

Study or Subgroup	Absence Events	Absence Total	Presence Events	Presence Total	Weight	Odds Ratio M-H, Fixed, 95% CI
Ishii et al., 2017	108	157	26	33	24.0%	0.59 [0.24, 1.46]
Iwaoka et al., 2014	51	63	18	23	9.0%	1.18 [0.37, 3.82]
Iwaoka et al., 2016	48	61	14	20	8.0%	1.58 [0.51, 4.93]
Okazaki et al., 2013	158	217	8	13	7.3%	1.67 [0.53, 5.32]
Tomioka et al., 2019	235	353	53	80	51.7%	1.01 [0.61, 1.70]
**Total (95% CI)**		**851**		**169**	**100.0%**	**1.02 [0.71, 1.48]**
Total events	600		119			

Heterogeneity: $Chi^2 = 2.73$, $df = 4$ ($P = 0.60$); $I^2 = 0\%$

Test for overall effect: $Z = 0.12$ ($P = 0.91$)

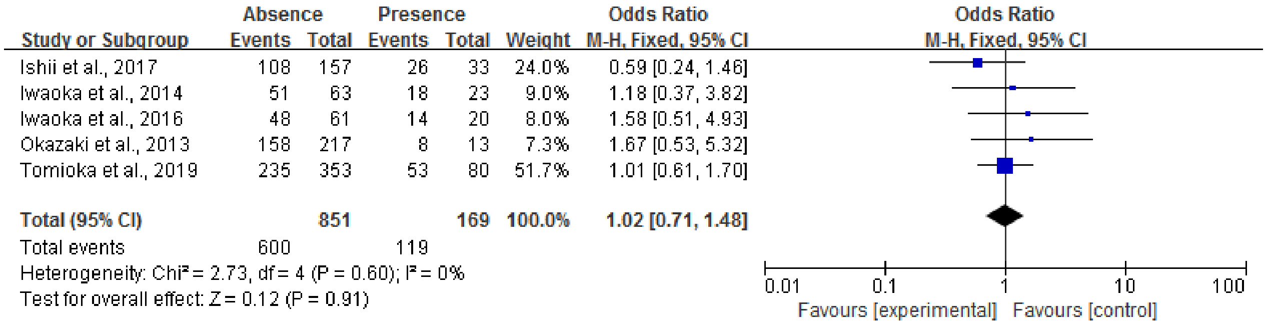

Figure 31. Funnel plot of having diabetes at 12 weeks

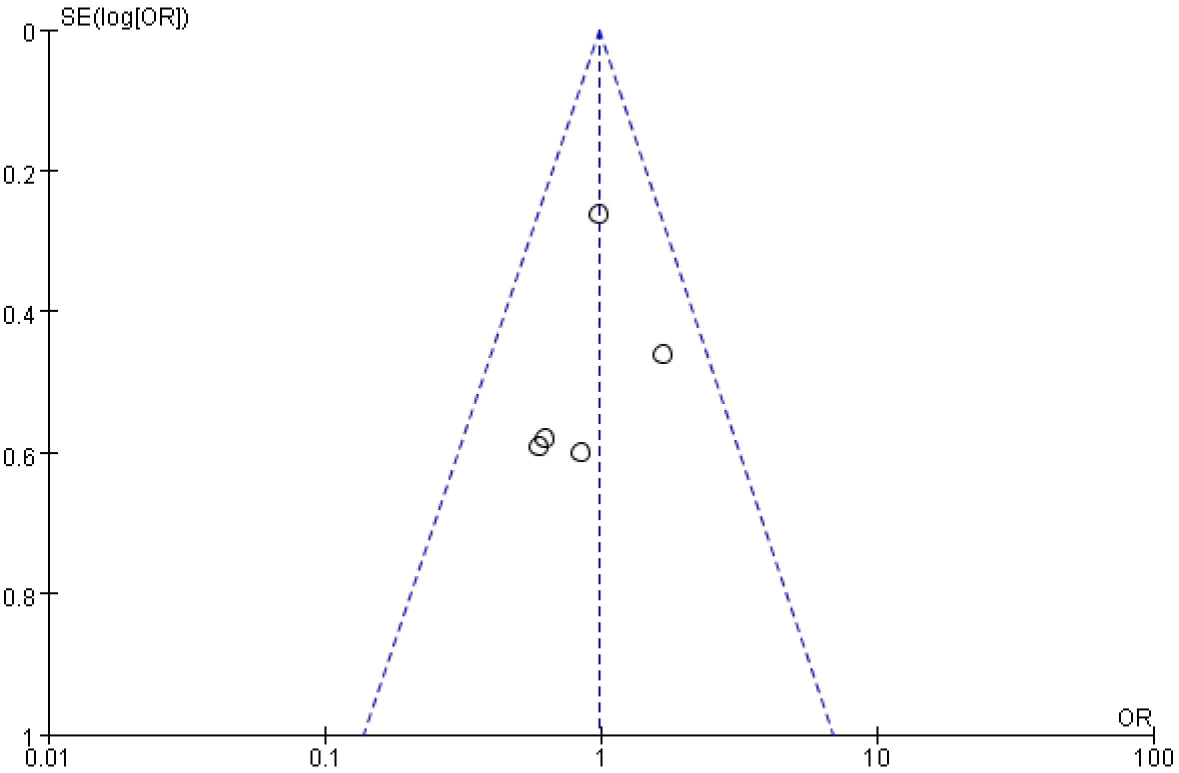

Figure 32. Forest plot of having hypertension at 12 weeks

Study or Subgroup	Absence Events	Absence Total	Presence Events	Presence Total	Weight	Odds Ratio M-H, Fixed, 95% CI
Ishii et al., 2017	98	145	36	45	24.2%	0.52 [0.23, 1.17]
Iwaoka et al., 2014	38	45	31	41	6.9%	1.75 [0.60, 5.14]
Iwaoka et al., 2016	36	44	26	37	7.0%	1.90 [0.67, 5.39]
Okazaki et al., 2013	148	208	18	22	12.8%	0.55 [0.18, 1.69]
Tomioka et al., 2019	221	338	67	95	49.2%	0.79 [0.48, 1.29]
**Total (95% CI)**		**780**		**240**	**100.0%**	**0.84 [0.60, 1.18]**
Total events	541		178			

Heterogeneity: Chi² = 6.12, df = 4 (P = 0.19); I² = 35%

Test for overall effect: Z = 1.02 (P = 0.31)

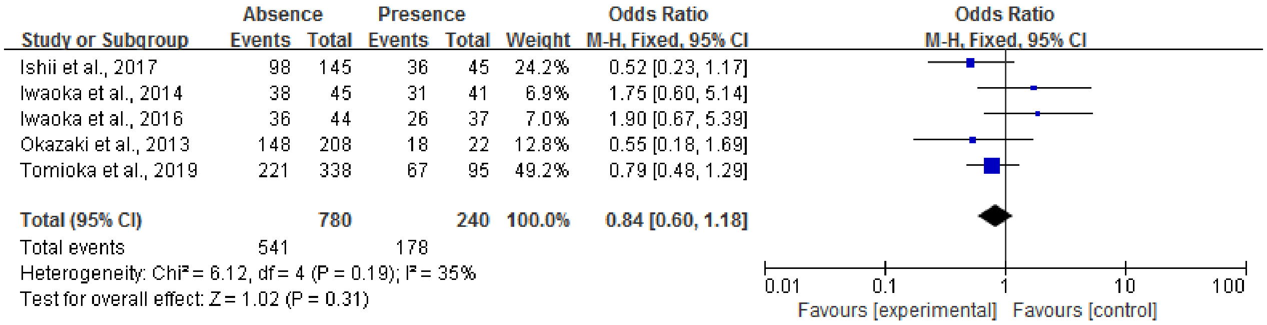

Figure 33. Forest plot of having hypertension at 12 weeks

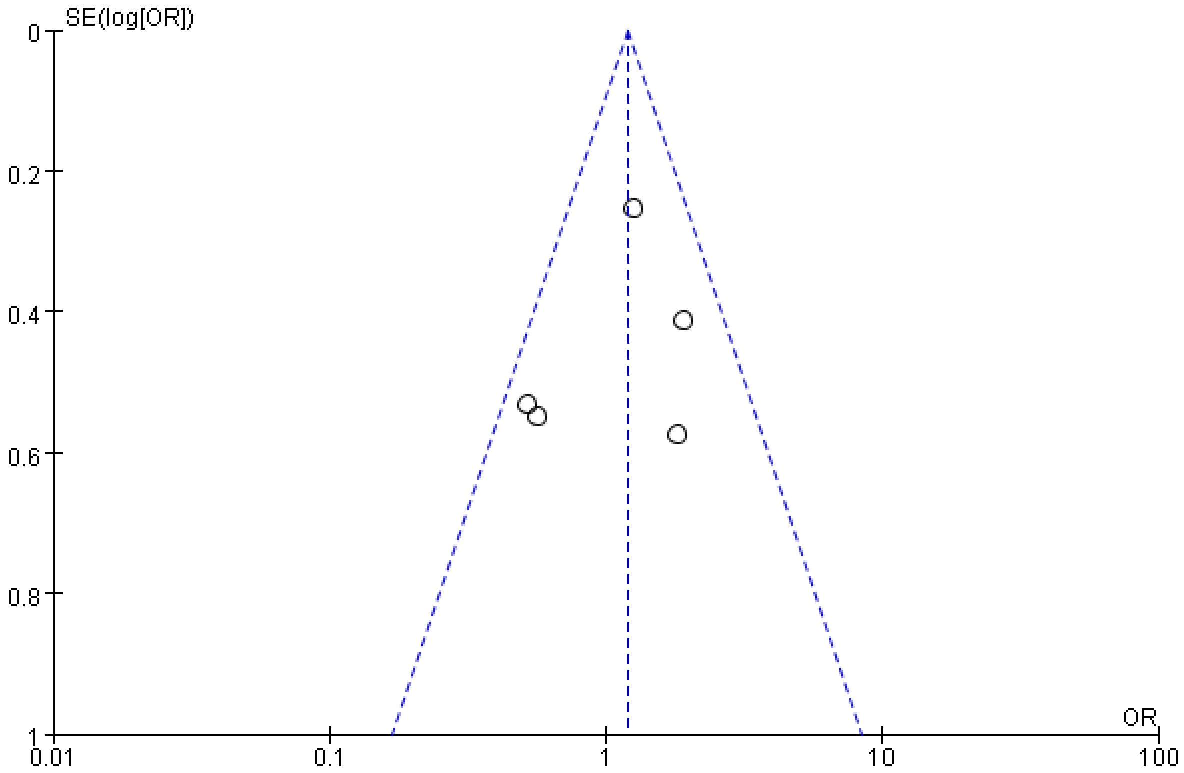

Figure 34. Forest plot of the CO concentration of the breath at the start of treatment at 12 weeks

Study or Subgroup	Success group Mean	SD	Total	Failure group Mean	SD	Total	Weight	Mean Difference IV, Random, 95% CI
Itakura et al., 2016	28	14.4	44	19.7	13.7	37	17.7%	8.30 [2.17, 14.43]
Miyagi et al., 2012	20.7	12.1	69	22.8	13.2	18	16.6%	-2.10 [-8.83, 4.63]
Nobata et al., 2013	16.9	12	78	17.8	11	34	20.6%	-0.90 [-5.46, 3.66]
Sugiyama, 2018	17.6	16.1	157	20	16.3	73	20.6%	-2.40 [-6.91, 2.11]
Tomioka et al., 2019	13.3	8.6	288	19.5	10.9	145	24.5%	-6.20 [-8.23, -4.17]
**Total (95% CI)**			**636**			**307**	**100.0%**	**-1.08 [-5.70, 3.55]**

Heterogeneity: Tau² = 21.70; Chi² = 22.48, df = 4 (P = 0.0002); I² = 82%
Test for overall effect: Z = 0.46 (P = 0.65)

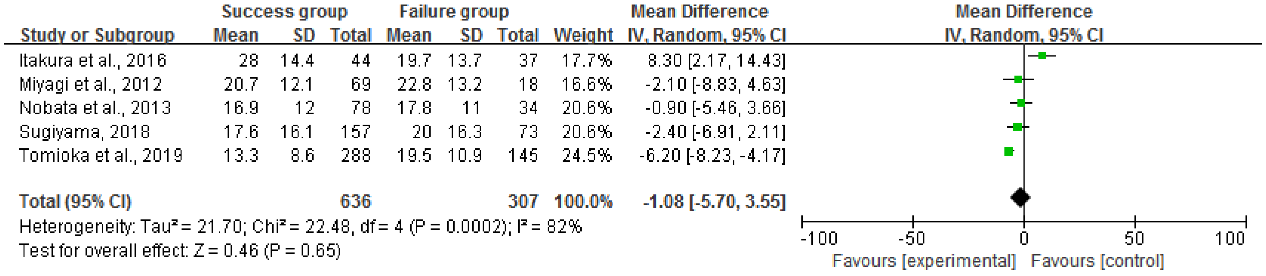

Figure 35. Funnel plot of the CO concentration of the breath at the start of treatment at 12 weeks

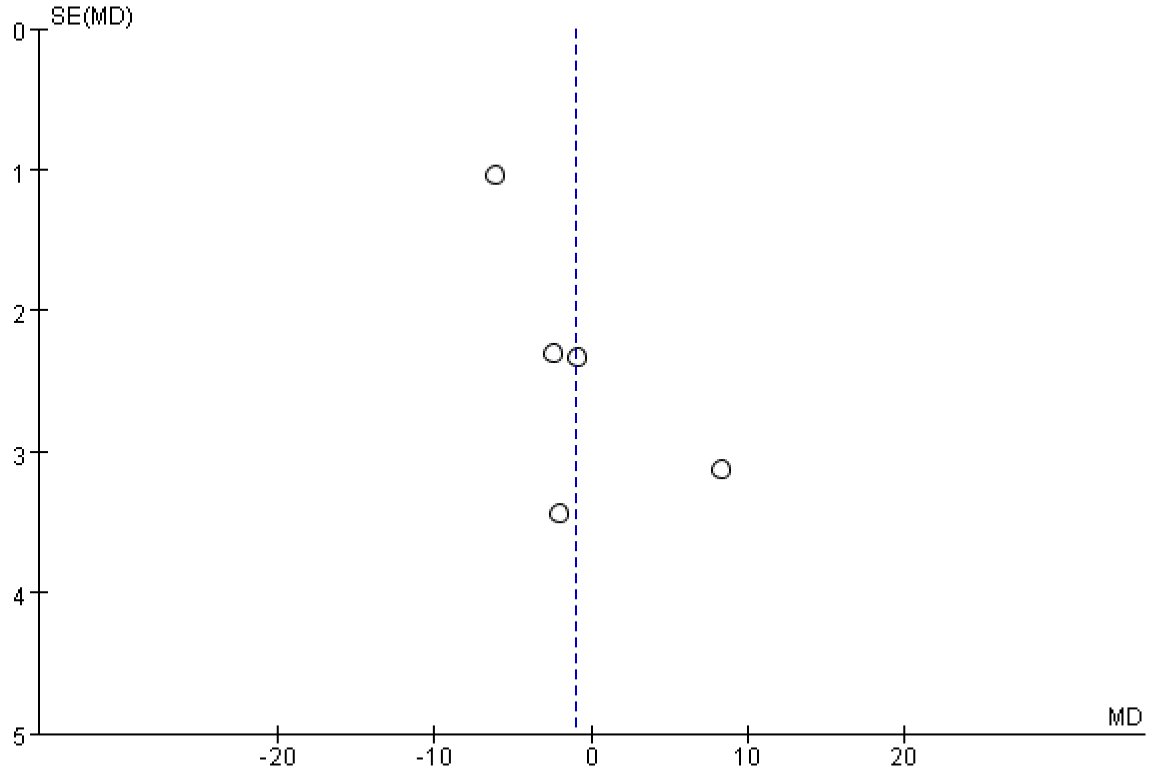

Figure 36. Forest plot of cohabitant with smokers at 12 weeks

Study or Subgroup	Absence Events	Absence Total	Presence Events	Presence Total	Weight	Odds Ratio M-H, Random, 95% CI
Itakura et al., 2016	32	61	12	20	17.7%	0.74 [0.26, 2.05]
Nobata et al., 2013	54	64	33	69	20.3%	5.89 [2.59, 13.42]
Sawayama et al., 2011	39	51	8	13	14.6%	2.03 [0.56, 7.39]
Taniguchi et al., 2017	404	957	123	328	26.6%	1.22 [0.94, 1.58]
Uchida, 2007	47	64	24	52	20.9%	3.23 [1.48, 7.02]
**Total (95% CI)**		**1197**		**482**	**100.0%**	**2.02 [0.99, 4.16]**
Total events	576		200			

Heterogeneity: Tau² = 0.49; Chi² = 18.89, df = 4 (P = 0.0008); I² = 79%
Test for overall effect: Z = 1.92 (P = 0.05)

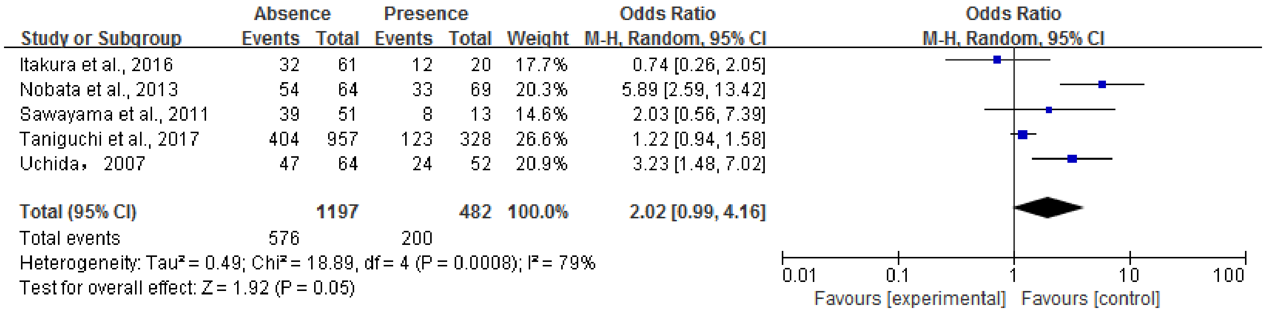

Figure 37. Forest plot of cohabitant with smokers

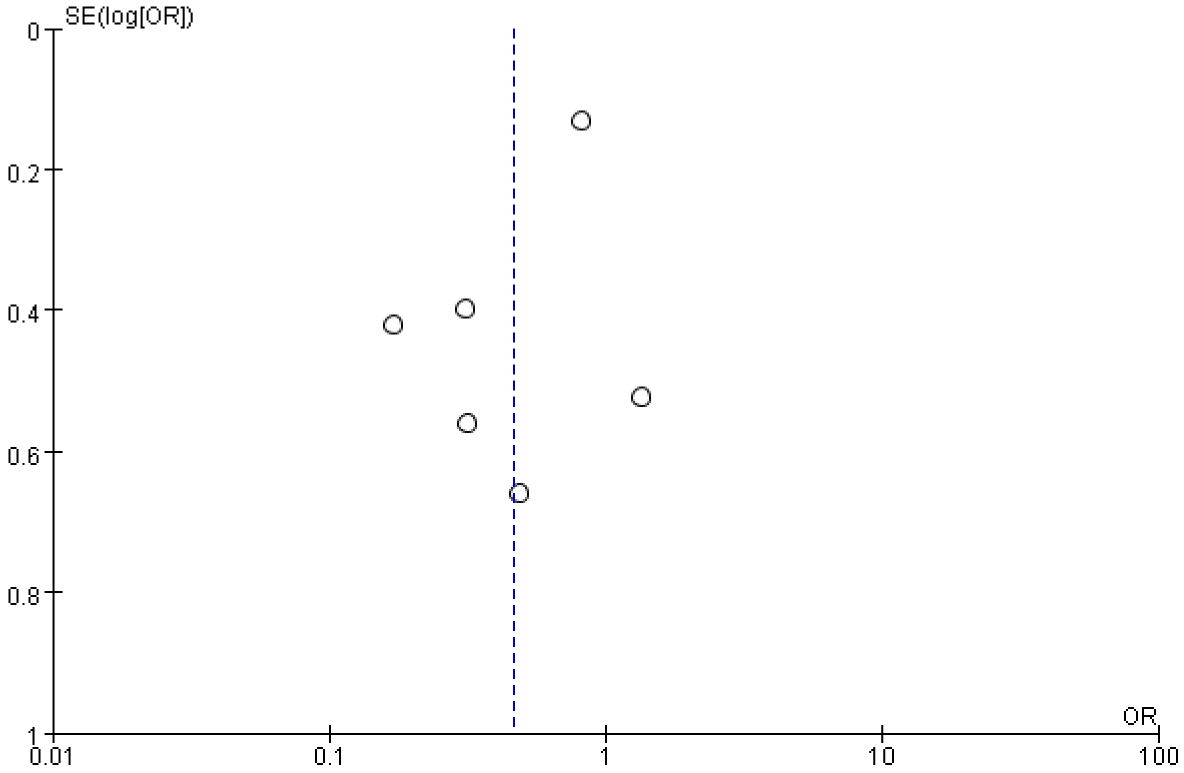

Figure 38. Forest plot of previous abstinence at 1 year

Study or Subgroup	Experience Events	Experience Total	Not experience Events	Not experience Total	Weight	Odds Ratio M-H, Fixed, 95% CI
Kurioka et al., 2009	16	39	6	22	5.8%	1.86 [0.60, 5.77]
Miyagi et al., 2012	31	52	23	32	14.7%	0.58 [0.22, 1.49]
Sawayama et al., 2011	11	27	21	36	13.6%	0.49 [0.18, 1.35]
Taniguchi et al., 2017	192	419	56	114	60.9%	0.88 [0.58, 1.33]
Taniguchi(H) et al., 2018	21	31	7	7	5.0%	0.14 [0.01, 2.62]
**Total (95% CI)**		**568**		**211**	**100.0%**	**0.80 [0.57, 1.11]**
Total events	271		113			

Heterogeneity: Chi² = 5.01, df = 4 (P = 0.29); I² = 20%

Test for overall effect: Z = 1.33 (P = 0.18)

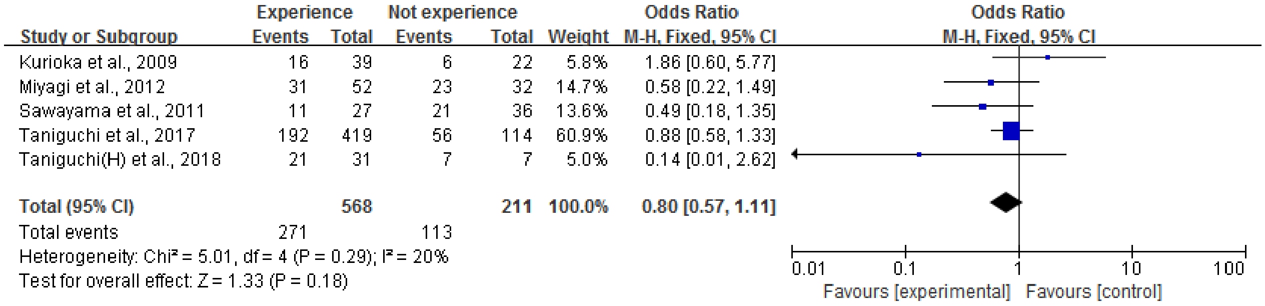

Figure 39. Funnel plot of previous abstinence

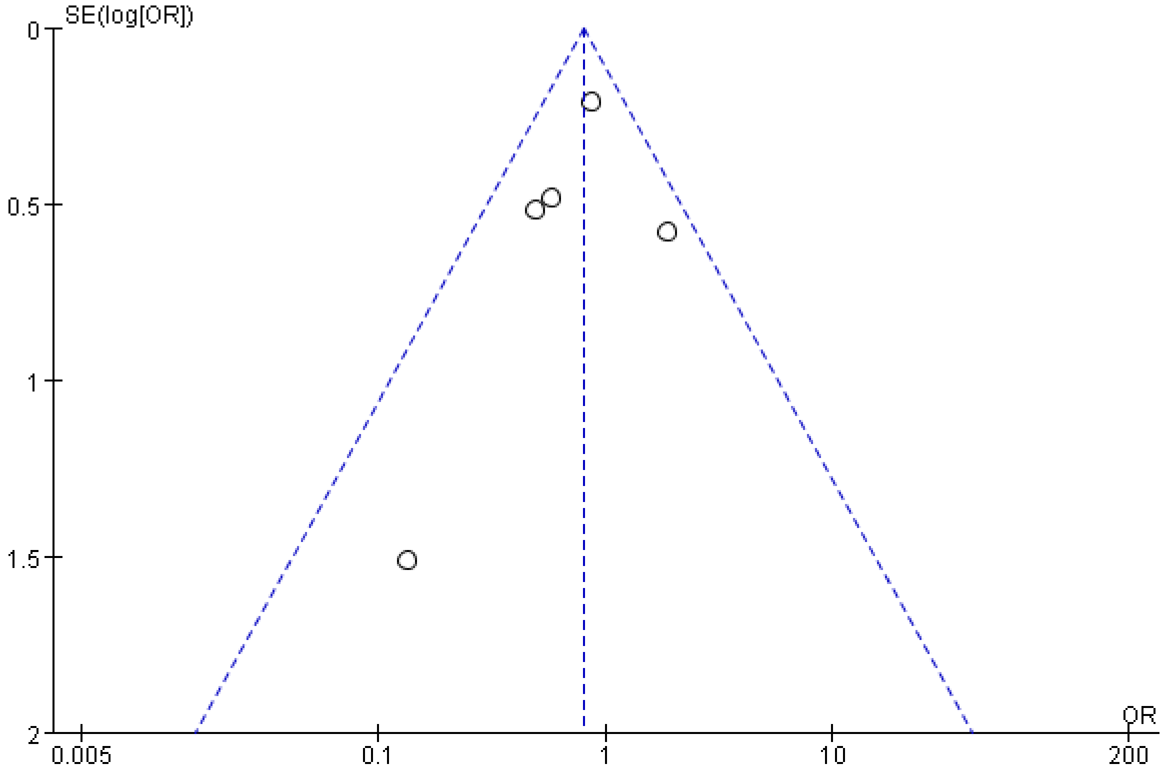

Meta-analysis

To assess the effect of gender differences on smoking cessation among the studies included in systematic review, we conducted meta-analysis to evaluate the effect of gender differences on smoking cessation. For meta-analysis, the results of meta-analysis are reported as odds ratio (OR) with 95% confidence interval (CI). Chi-squared heterogeneity test (*p*-value) and Higgins index (I^2) are applied to evaluate the heterogeneity among included articles. Heterogeneity is regarded as: none (I^2 <25%), low (25%≤ I^2 <50%), moderate (25%≤ I^2 <75%), or high (I^2 ≥ 75%). The fixed effect model is applied when slight heterogeneity (*p*-value<0.05 or I^2 ≤ 50%), otherwise, the random effect model is used. In addition, we generated and visually analyzed funnel plots for testimonies of publication bias. All analyses are conducted by the software RevMan (Review Manager, version 5.3).

References

1. Taniguchi C, Tanaka H, Saka H, et al. Cognitive, behavioural and psychosocial factors associated with successful and maintained quit smoking status among patients who received smoking cessation intervention with nurses' counselling. *J Adv Nurs.* 2017;73(7):1681-1695.

   doi: 10.1111/jan.13258.

2. Taniguchi C, Tanaka H, Saka H, et al. Changes in self-efficacy associated with success in quitting smoking in participants in Japanese smoking cessation therapy. *Int J Nurs Pract.*

   2018;24(4):e12647. doi: 10.1111/ijn.12647

3. Tomioka H, Wada T, Yamazoe M, Yoshizumi Y, Nishio C, Ishimoto G. Ten-year experience of

   smoking cessation in a single center in Japan. Respir Investig. 2019,

   pii: S2212-5345(18)30227-2. doi: 10.1016/j.resinv.2019.01.007.

4. Uchida K. Smoking cessation treatment, analysis of smoking cessation rate, and the reason of lower smoking cessation rate in women compared with that in men. *Nihon Kokyuki Gakkai Zasshi.* 2007;45(9):673-678. (In Japanese)

5. Makiko Yamamoto. The quit rate of tobacco smoking treated by the smoking cessation specialist admitted by Japanese Society Tobacco Control. *Japanese journal of tobacco control.* 2007;2(8):126-129. (In Japanese)

6. Kurioka N, Morooka Y, Yoshii C, Inagaki K, Sezai I, Kano M. Efficacy and ongoing problems in a three-month smoking cessation program established under the public health insurance system in Japan. *Japanese journal of tobacco control.* 2008;3(1):3-11. (In Japanese)

7. Ito A , Ito H, Miura H, Takahashi Y. Examination of factors influencing the short- and long-term success rate (1-year smoking cessation rate) of smoking cessation treatment with a nicotine patch. *Smoking cessation science.* 2008;2(2):17-22. (In Japanese)

8. Hirata A, Sato S, Nagatomi H, Tamagaki Y. Our experience of using varenicline (Champix ®). *Japanese journal of tobacco control.* 2009;4(2):27-32. (In Japanese)

9. Tanaka M, Muta K, Iwatubo H. High Success Rate in Smoking Cessation Due to Cooperative Team Intervention. *Official Journal of Japan Society of Ningen Dock.* 2010;25(1):100-104. DOI:https://doi.org/10.11320/ningendock.25.100 (In Japanese)

10. Sawayama T, Shibata T, Murakami T, et al. Examination of factors affecting short-term and long-term smoking cessation rate. *The Teishin igaku.* 2011;63(1):48-55. (In Japanese)

11. Taniguchi C , Tanaka H, Itakura A, et al. Factors associated with 4-weeks quit rate before the end of smoking cessation therapy in Japan. *Japanese journal of tobacco control.* 2011;6(3):34-39. (In Japanese)

12. Sato T, Ohkawa T, Soma S. Achievement point of Kyoritsu clinic "smoking cessation outpatient" —Results and themes—. *Aomori Kyoritsu Hospital Medical Journal.* 2013;7:9-12. (In Japanese)

13. Satoh K, Seiji K, Mizoguchi K, Igarashi T, Matsui K. Smoking cessation rate and smoking persistence rate by gender in JR Sendai Hospital outpatient clinic. *Japanese journal of tobacco control*. 2012;7(4):123-127. (In Japanese)

14. Miyagi O, Toyosato T, Kobayashi S, Kawaguchi A. Epidemiological study for long lasting effect of smoking cessation treatment: follow up study during 3 months and a year. *Journal of the Human Arts and Sciences.* 2012;8(2):91-99. DOI:https://doi.org/10.11427/jhas.8.91 (In Japanese)

15. Okazaki S, Ueda K. Smoking cessation using varenicline: Relation between the abstinence rate and the age and gender. *Annals of The Japanese Respiratory Society.* 2013;2(4):327-332. (In Japanese)

16. Yoshii C, Nishida C, Kawanami Y, et al. Evaluation of the 12-week treatment results by varenicline (Champix ®). *Japanese journal of tobacco control.* 2013;8(1):13-20. (In Japanese)

17. Nobata S, Nishiwaki R, Shimokuri E, et al. Examination of Results of Smoking Cessation Treatment with Nicotine Patches or Varenicline in Ningen Dock or Health Check-up Subjects and Factors Influencing those Results. *Official Journal of Japan Society of Ningen Dock.* 2013;27(5):843-850. DOI: https://doi.org/10.11320/ningendock.27.843 (In Japanese)

18. Taniguchi C, Tanaka H, Oze I, et al. Factors associated with weight gain after smoking cessation therapy in Japan. *Nurs Res.* 2013;62(6):414-421.

doi: 10.1097/NNR.0000000000000000.

19. Iwaoka M, Shimamura H, Tsuji T, Kugiyama K. Twelve weeks of smoking cessation therapy with Varenicline increases the serum levels of apolipoprotein A-I only in the success group.

J Cardiol. 2014;64(4):318-323. doi: 10.1016/j.jjcc.2014.02.009.

20. Yamazaki Y. Invention of support to influence the success rate of smoking cessation. *Collection of Hokkaido Nursing Research Association 15.* 2015:58-60.

21. Yasuda M, Suzuki A, Takahashi A, et al. Analysis of factors associated with successful smoking cessation and conditions abstinence after smoking cessation therapy at our clinic. *Health Evaluation and Promotion.* 2015;42(3):385-391. DOI:https://doi.org/10.7143/jhep.42.385 (In Japanese)

22. Iwaoka M, Tsuji T. Twelve Weeks of Successful Smoking Cessation Therapy with Varenicline Reduces Spirometric Lung Age. *Intern Med.* 2016;55:2387-2392.

DOI: https://doi.org/10.2169/internalmedicine.55.6844

23. Shimadu S, Hamajima N, Okada Y, et al. Factors influencing sustainable efficacy of smoking cessation treatment with varenicline beyond nine months. *Nagoya J Med Sci.* 2016;78(2):205-213.

24. Itakura Y, Saito E, Muramatsu H. Relationships between self-efficacy and continuation of smoking cessation among outpatients visiting a clinic for smoking cessation. *Japanese journal of tobacco control.* 2016;11(2):22-30.

DOI: https://doi.org/10.14950/jstc.11.22 (In Japanese)

25. Ishii M, Ohnishi T, Morisaki K, et al. Clinical response to varenicline as a smoking cessation aid. *Japanese journal of tobacco control.* 2017;12(3):58-63.

DOI:https://doi.org/10.14950/jstc.12.58 (In Japanese)

26. Taniguchi H, Yoshii C, Isomura T, et al. Changes in psychological factors following nine months of abstinence after attending a smoking cessation clinic. *Annals of The Japanese Respiratory Society.* 2018;7(1):2-9. (In Japanese)

27. Sugiyama M. Analysis of factors associated with varenicline therapeutic efficacy at three different hospitals. *Japanese journal of tobacco control.* 2018;13(4):64-70. DOI:https://doi.org/10.14950/jstc.13.64 (In Japanese)

28. Kurioka N, Hirota I, Yoshii C, Inagaki K, Sezai I, Kano M. Smoking cessation rate one-year after smoking cessation program and evaluation of smoker's perception of smoking using the KTSND (The Kano Test for Social Nicotine Dependence) questionnaire. *Japanese journal of tobacco control.* 2009;4(1):3-11. (In Japanese)

© 2020 Li M. and Okamoto R.
